# Placental galectins regulate innate and adaptive immune responses in pregnancy

**DOI:** 10.3389/fimmu.2022.1088024

**Published:** 2022-12-28

**Authors:** Orsolya Oravecz, Roberto Romero, Eszter Tóth, Judit Kapitány, Máté Posta, Dahiana M. Gallo, Simona W. Rossi, Adi L. Tarca, Offer Erez, Zoltán Papp, János Matkó, Nándor Gábor Than, Andrea Balogh

**Affiliations:** ^1^ Systems Biology of Reproduction Research Group, Institute of Enzymology, Research Centre for Natural Sciences, Budapest, Hungary; ^2^ Doctoral School of Biology, Institute of Biology, ELTE Eötvös Loránd University, Budapest, Hungary; ^3^ Perinatology Research Branch, Eunice Kennedy Shriver National Institute of Child Health and Human Development, National Institutes of Health, United States Department of Health and Human Services, Detroit, MI, United States; ^4^ Department of Obstetrics and Gynecology, University of Michigan, Ann Arbor, MI, United States; ^5^ Department of Epidemiology and Biostatistics, Michigan State University, East Lansing, MI, United States; ^6^ Center for Molecular Medicine and Genetics, Wayne State University, Detroit, MI, United States; ^7^ Detroit Medical Center, Detroit, MI, United States; ^8^ Károly Rácz Doctoral School of Clinical Medicine, Semmelweis University, Budapest, Hungary; ^9^ Department of Obstetrics and Gynecology, Wayne State University, Detroit, MI, United States; ^10^ Department of Obstetrics and Gynecology, Universidad Del Valle, Cali, Colombia; ^11^ Genesis Theranostix Group, Budapest, Hungary; ^12^ Department of Obstetrics and Gynecology, Soroka University Medical Center, Beer Sheva, Israel; ^13^ Department of Obstetrics and Gynecology, Semmelweis University, Budapest, Hungary; ^14^ Maternity Private Clinic of Obstetrics and Gynecology, Budapest, Hungary

**Keywords:** danger signal, evolution, glycomics, leukocyte, obstetrical syndrome, PP13, primate, trophoblast

## Abstract

**Introduction:**

Galectins are master regulators of maternal immune responses and placentation in pregnancy. Galectin-13 (gal-13) and galectin-14 (gal-14) are expressed solely by the placenta and contribute to maternal-fetal immune tolerance by inducing the apoptosis of activated T lymphocytes and the polarization of neutrophils toward an immune-regulatory phenotype.Furthermore, their decreased placental expression is associated with pregnancy complications, such as preeclampsia and miscarriage. Yet, our knowledge of the immunoregulatory role of placental galectins is incomplete.

**Methods:**

This study aimed to investigate the effects of recombinant gal-13 and gal-14 on cell viability, apoptosis, and cytokine production of peripheral blood mononuclear cells (PBMCs) and the signaling pathways involved.

**Results:**

Herein, we show that gal-13 and gal-14 bind to the surface of non-activated PBMCs (monocytes, natural killer cells, B cells, and T cells) and increase their viability while decreasing the rate of their apoptosis without promoting cell proliferation. We also demonstrate that gal-13 and gal-14 induce the production of interleukin (IL)-8, IL-10, and interferon-gamma cytokines in a concentration-dependent manner in PBMCs. The parallel activation of Erk1/2, p38, and NF-ĸB signaling evidenced by kinase phosphorylation in PBMCs suggests the involvement of these pathways in the regulation of the galectin-affected immune cell functions.

**Discussion:**

These findings provide further evidence on how placenta-specific galectins assist in the establishment and maintenance of a proper immune environment during a healthy pregnancy.

## Introduction

The interactions of maternal, fetal, and placental immune responses during pregnancy as well as the mechanisms that maintain maternal immune tolerance to the semi-allogeneic fetus, while guarding against microbial infection, are of great interest in reproductive medicine, even though these topics have been only partially explored (1–12). Investigators have described that time- and site-specific modifications are characteristic of these immune interactions, such as the pro-inflammatory milieu at the time of implantation, the anti-inflammatory state during the second trimester, and local and systemic inflammation in the third trimester, preceding delivery (13–17). The mediators of this complex network of maternal-fetal-placental immune interactions involve a large array of cellular, vesicular, and soluble components (8, 18–29). In the past decade, an increasing body of evidence has been collected on the key contribution of galectins to immune interactions in pregnancy (30–43).

Galectins are glycan-binding proteins belonging to the lectin subfamily, with the common property of specifically binding to glycoconjugates containing *β*-galactoside carbohydrates (9, 20, 44). Of the 19 galectins found in mammals, 13 are expressed in human tissues (20, 44–46). These human galectins affect a variety of cellular processes when they contact immune cells, e.g., apoptosis, cell proliferation, cell adhesion, chemotaxis, cytokine production, or degranulation (31, 39, 41, 47–52). In pregnancy, several galectins are expressed at the maternal-fetal interface and assist in regulating placental and fetal development, local and systemic inflammation as well as the establishment and maintenance of maternal immune tolerance toward the fetus (9, 38, 46, 48, 49, 53–58).

In humans, three galectins (gal-13, gal-14, and gal-16) are expressed solely by the placenta (20, 46, 57, 59). The genes encoding these galectins are located within a cluster of galectin genes and pseudogenes on chromosome 19, which arose in anthropoid primates through birth-and-death evolution (20, 46, 59, 60). This evolutionary gene duplication process led to neofunctionalization, the acquisition of a new function(s) in the duplicated genes, as measured by the differing carbohydrate-binding capacity of these placental galectins (59). At the RNA and protein levels, these are primarily expressed in the multinucleated syncytiotrophoblast layer of the placenta, formed by the fusion of mononucleated cytotrophoblast cells, but not in the underlying progenitor cytotrophoblasts (59, 61). In comparison, expression of these galectins is less abundant in the extravillous trophoblasts, amniotic epithelium, and fetal endothelium (53, 59, 60, 62).

During a healthy pregnancy, maternal blood gal-13 concentrations increase with advancing gestation and then diminish after delivery (61, 63). Notably, gal-13 is secreted into the maternal circulation from the syncytiotrophoblast (62–65), and low gal-13 concentrations were found in the maternal circulation during the first trimester in women who later developed preterm preeclampsia (66–70), a severe obstetrical syndrome with a strong systemic immune dysregulation (19, 71–76). In line with these findings, we have also demonstrated that the placental expression of gal-13 and gal-14 is down-regulated in preterm preeclampsia (60, 62, 64, 77) and in miscarriage (78).

Although several galectins have been studied extensively regarding their effects on maternal immune cells (79–85), placenta-specific galectins have been less studied in this context. We and others have explored that gal-13 and gal-14 treatment induces apoptosis of activated T cells (59, 78) and alters the cell surface expression of T-cell activation markers (78). On the other hand, gal-13 induces the production of interleukin (IL)-1ɑ and IL-6 in peripheral blood mononuclear cells (PBMC) (86) and the production of IL-8 in non-activated T cells (78), but decreases tumor necrosis factor-alpha expression in neutrophils (87). Overall, gal-13 may have a critical role in the maintenance of the physiological immune balance at the maternal-fetal interface and its lower expression may contribute to immune dysregulation in pregnancy complications such as preeclampsia, intrauterine growth restriction, and miscarriage. This phenomenon was also suggested for other galectins expressed at the maternal-fetal interface such as gal-1 and gal-3, highlighting their importance in proper placental functions (20, 36, 43, 55, 86, 88–91).

To further characterize the role of placenta-specific galectins in the regulation of maternal-fetal immune interactions, we aimed to investigate the effects of recombinant gal-13 and gal-14 on the viability, apoptosis, cytokine production, and cell signaling of PBMCs.

## Materials and methods

### Expression and purification of recombinant gal-13 and gal-14

Recombinant gal-13 and gal-14 were expressed in *ClearColi BL21*(DE3) (Lucigen, Middleton, WI, USA) bacterial strains with slight modifications, as previously described by Balogh et al. (78). All purification steps were carried out in the presence of 1mM dithiothreitol (DTT). Gal-13 and gal-14 were aliquoted in phosphate-buffered saline (PBS; Thermo Fisher Scientific, Waltham, MA, USA) supplemented with 1 mM DTT and stored at −80°C until use.

### Isolation of peripheral blood mononuclear cells

Buffy coats, obtained from healthy, non-pregnant human females (n=3-5, depending on the experiment), were purchased from the Hungarian National Blood Transfusion Service (Budapest, Hungary). PBMCs were isolated by Ficoll-Paque PLUS (GE Healthcare Life Sciences, Chicago, IL, USA) density gradient centrifugation. Isolated cells were washed with PBS before the experiments. PBMCs were placed into RPMI-1640 medium (Thermo Fisher Scientific) supplemented with 10% fetal bovine serum (FBS; Thermo Fisher Scientific) and gentamicin (Thermo Fisher Scientific). Cell counts were determined with trypan blue stain and a TC20 automated cell counter (Bio-Rad, Hercules, CA, USA). Isolated PBMCs were cultured for 24–72 hours depending on the experiment. To obtain experimental replicates, all assays were repeated with multiple donors.

### Binding of recombinant gal-13 and gal-14 to peripheral blood mononuclear cells

Recombinant gal-13 or gal-14, conjugated with CF488 (Mix-n-Stain CF488 Kit, Biotium, Fremont, CA, USA), according to the manufacturer’s instructions, was added to 5×10^5^ PBMCs in PBS containing 0.5% bovine serum albumin (BSA, Sigma-Aldrich, St. Louis, MO, USA) and 0.05% sodium azide (flow cytometry wash buffer), and incubated on ice for 45 minutes. Samples were washed with wash buffer at 500*g* for 5 minutes at 4°C. To reduce nonspecific antibody binding, Fc receptors were blocked with human FcR blocking reagent (Miltenyi Biotec, Bergisch Gladbach, Germany) for 5 minutes on ice. Population marker antibodies (**Supplementary Table 1**) were added to the cells, then incubated for 20 minutes on ice in the dark. Cells were then washed twice and resuspended in wash buffer. Flow cytofluorimetric measurements were carried out on a CytoFLEX device and with CytExpert software (Beckman Coulter, Brea, CA, USA) for data acquisition. Data were collected from 50,000 cells/sample and analyzed with FlowJo v10 software (FlowJo LLC, Ashland, OR, USA).

### Cell viability assay

PBMCs (2×10^5^ cells/well) were treated with 0.04 µM, 0.4 µM, or 4 µM recombinant gal-13, gal-14, or vehicle (PBS supplemented with 1 mM DTT in the same dilution) and incubated on 96-well plates in RPMI-1640 medium supplemented with 10% FBS and gentamicin for 68 hours (at 37°C, 5% CO_2_). CCK-8 solution (Sigma-Aldrich) was added to the wells, and the plates were incubated for 4 hours at 37°C. Absorbance at 450 nm was measured by using an EnSpire microplate reader (Perkin Elmer, Waltham, MA, USA).

### Apoptosis assay

PBMCs (4×10^5^ cells/well) were treated with 4 µM of recombinant gal-13 or gal-14. Galectin-treated and vehicle-treated cells were incubated for 24 hours on 24-well tissue culture plates (Eppendorf, Hamburg, Germany) in RPMI-1640 medium supplemented with 10% FBS and gentamicin. PBMCs were transferred to flow cytometry tubes and washed with wash buffer at 350g for 10 minutes at 4°C. Then, cells were stained with population marker antibodies (**Supplementary Table 1**) to discriminate between PBMC populations. After incubation on ice for 20 minutes in the dark, cells were washed twice in wash buffer. To differentiate between early-stage and late-stage apoptotic cells, phycoerythrin-conjugated annexin V (annexin V-PE) and 7-aminoactinomycin D (7-AAD) were applied in the presence of annexin binding buffer (Annexin-V/7-AAD Apoptosis Detection Kit, BioLegend, San Diego, CA, USA). After incubation for 15 minutes in the dark at room temperature, annexin binding buffer was added, and samples were measured immediately on a CytoFLEX flow cytometer. Data were collected from 30,000 cells/sample and analyzed by using FlowJo v10 software (FlowJo, LLC).

### Measurement of cell proliferation by flow cytometry

PBMCs (4×10^6^ cells/ml) were incubated with carboxyfluorescein succinimidyl ester (CFSE) cell staining dye (BioLegend) for 20 minutes at room temperature in the dark. Next, cells were incubated for 5 minutes at room temperature in RPMI-1640 medium supplemented with 10% FBS and gentamicin. Cells were then centrifuged twice with medium at 300g for 10 minutes at 20°C. Cell concentration was determined by trypan blue staining (Bio-Rad). Overall, 5×10^5^ cells were plated in 48-well cell culture plates (Eppendorf) in completed RPMI-1640 medium and treated with 4 μM of gal-13, gal-14, or vehicle for 72 hours (37°C, 5% CO_2_). Then, cells were centrifuged with PBS (350 g, 4°C, 8 minutes) and incubated for 20 minutes with Zombie Violet vitality dye (BioLegend) at room temperature. After centrifugation in wash buffer, PBMC populations were labeled with cell-specific antibodies (**Supplementary Table 1**). Cells were incubated for 20 minutes in the dark on ice, followed by the addition of wash buffer and centrifugation twice. Subsequently, cells were resuspended in wash buffer and measured with a CytoFLEX cytofluorimeter. Data were collected from 100,000 cells/sample, and the results were analyzed with FlowJo v10 software (FlowJo, LLC).

### Measurement of cytokine production by enzyme-linked immunosorbent assay

Commercially available Human ELISA Kits for IL-8, IL-10, and interferon-gamma (IFN-γ) (Biolegend) were used to estimate the concentration of cytokines in the culture medium (RPMI-1640 supplemented with 10% FBS and gentamicin) of PBMCs treated with 0.04 µM, 0.4 µM, or 4 µM of recombinant gal-13, gal-14, or vehicle for 24 hours. The procedure was carried out by following the manufacturer’s protocol. The reaction was stopped with 4N sulfuric acid and then the absorbance was measured at 450 nm (reference filter: 620 nm) with an EnSpire microplate reader (Perkin Elmer).

### Western blot for phospho-protein detection

PBMCs (6×10^5^ cells/sample) were incubated with recombinant gal-13 (4 µM), gal-14 (4 µM), vehicle, or cell culture medium (absolute control). Samples were incubated for 10, 20, or 30 minutes at 37° C in a thermoblock (Grant Instruments Ltd., Shepreth, Cambridgeshire, United Kingdom) at 300 rpm. At the end of the treatment, samples were centrifuged at 20,000g for 30 seconds, supernatants were discarded, and cell pellets were frozen in liquid nitrogen. Then, a mixture of PBS, reducing 2× Laemmli sample buffer (Bio-Rad), protease, and phosphatase inhibitors (Thermo Fisher Scientific), was prepared and added to the frozen samples that were then heat-denatured at 95°C for 5 minutes. The electrophoretic separation was performed on 4-20% SDS-polyacrylamide gel (Thermo Fisher Scientific) in Tris-glycine buffer. Following SDS-PAGE, samples were blotted onto nitrocellulose membranes (Bio-Rad), which were blocked for 1 hour in Tris-buffered saline supplemented with 0.05% of Tween-20 (Sigma-Aldrich) (TBST) and 5% of BSA. Next, membranes were incubated overnight with rabbit anti-human primary antibodies specific to phosphorylated proteins, such as extracellular signal-regulated kinase 1/2 (Erk1/2), mitogen-activated protein kinase (MAPK) p38, or nuclear factor kappa B (NF-κB) (Cell Signaling Technology, Danvers, MA, USA) at 4°C in TBST containing 0.5% of BSA (**Supplementary Table 2**). The next day, membranes were incubated with horseradish peroxidase (HRP)-conjugated goat anti-rabbit IgG secondary antibody for 1 hour in TBST containing 0.5% of BSA (Sigma-Aldrich). β-actin-specific antibody was used as a loading control. Membranes were placed in an iBind Western blot device (Thermo Fisher Scientific) and incubated for 2 hours and 30 minutes with mouse anti-human β-actin (Thermo Fisher Scientific) primary antibody and HRP-conjugated rabbit anti-mouse secondary antibody (Life Technologies, Waltham, MA, USA), according to the manufacturer’s protocol (**Supplementary Table 2**). SuperSignal Western Pico PLUS Chemiluminescent Substrate (Thermo Fisher Scientific) was used to visualize the protein bands, and the ChemiDoc imaging device (Bio-Rad) was utilized for band detection. The relative signal intensities of phospho-proteins and β-actin were analyzed with ImageLab software (Bio-Rad).

### Statistical analysis

The one-way analysis of variance test with Tukey’s *post-hoc* test was used to compare cell viability/proliferation, apoptosis, and cytokine production of various galectin- and vehicle-treated groups. The one-sample t-test was used to analyze Western blot experiments. All analyses were performed with GraphPad Prism 5 software (GraphPad Software, San Diego, CA, USA). To ensure the concentration-dependent effect of recombinant galectins, we performed ordered factor analysis in R environment, using linear models, in case of cell viability and cytokine production experiments. Results were considered statistically significant at p<0.05.

## Results

### Gal-13 and gal-14 bind differentially to T and B lymphocytes, natural killer cells, and monocytes

**Figure 1:**
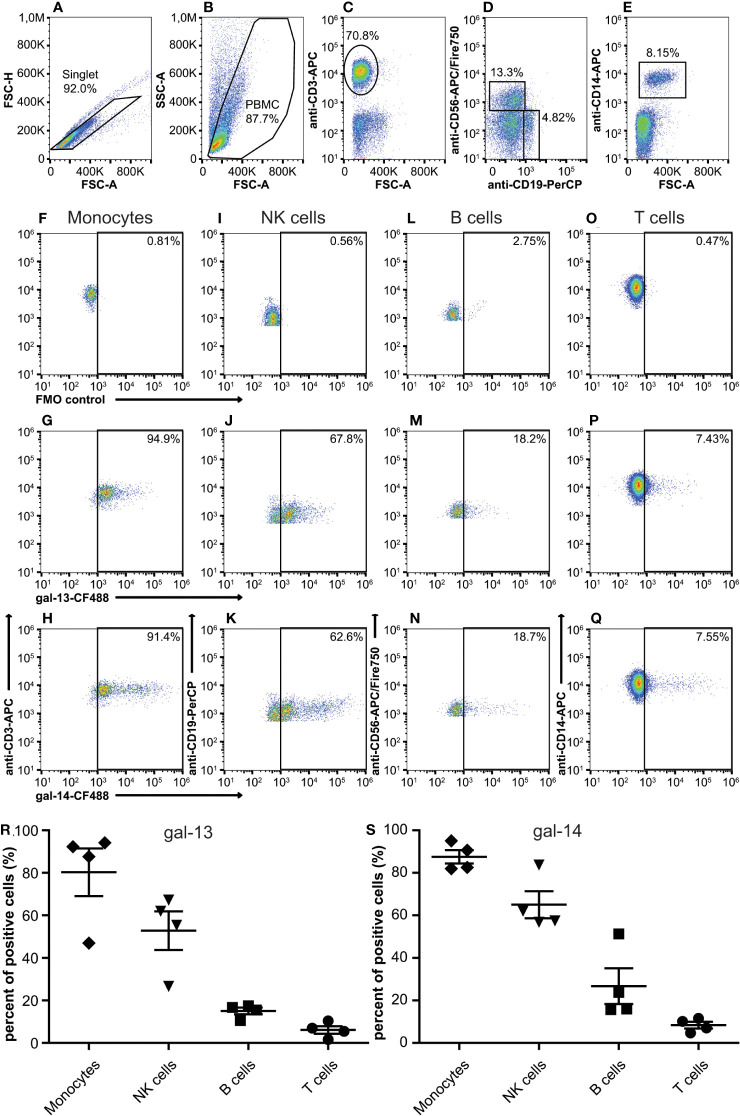
Binding of gal-13 and gal-14 to the peripheral blood mononuclear cells. Detection of gal-13 or gal-14 binding to human PBMCs was achieved by flow cytometry. Cells were incubated with recombinant gal-13-CF488 or gal-14-CF488. PBMCs were also stained with anti-CD3-APC, or anti-CD19-PerCP, anti-CD14-APC, and anti-CD56-APC/Fire750. Gating strategy **(A–Q)**. Graphs show the percentage of cells to which gal-13 **(R)** or gal-14 **(S)** bound (mean ± SEM, n = 4). FMO, Fluorescence minus one; PBMC, peripheral blood mononuclear cells; SEM, standard error of the mean.

The binding of gal-13 and gal-14 to helper and cytotoxic T cells as well as to neutrophil granulocytes has already been demonstrated by flow cytometry (78, 87). Herein, we extended these observations by examining the binding of fluorescent gal-13 and gal-14 to other PBMC populations as well, including B cells, natural killer (NK) cells, and monocytes (**Figures 1A–Q**). Recombinant gal-13 and gal-14 bound to the investigated PBMC populations to a different extent. The strongest binding of both galectins to monocytes (**Figures 1F–H, R, S**) was observed (47-95%), followed by NK cells (27-83%) and B cells (11-51%) (**Figures 1I–K, L–N, R, S**, respectively). Gal-13 and gal-14 bound the least to T cells (2-12%) (**Figures 1O–Q, R, S**).

### Gal-13 and gal-14 increase cell viability of immune cells through decreased apoptosis

**Figure 2:**
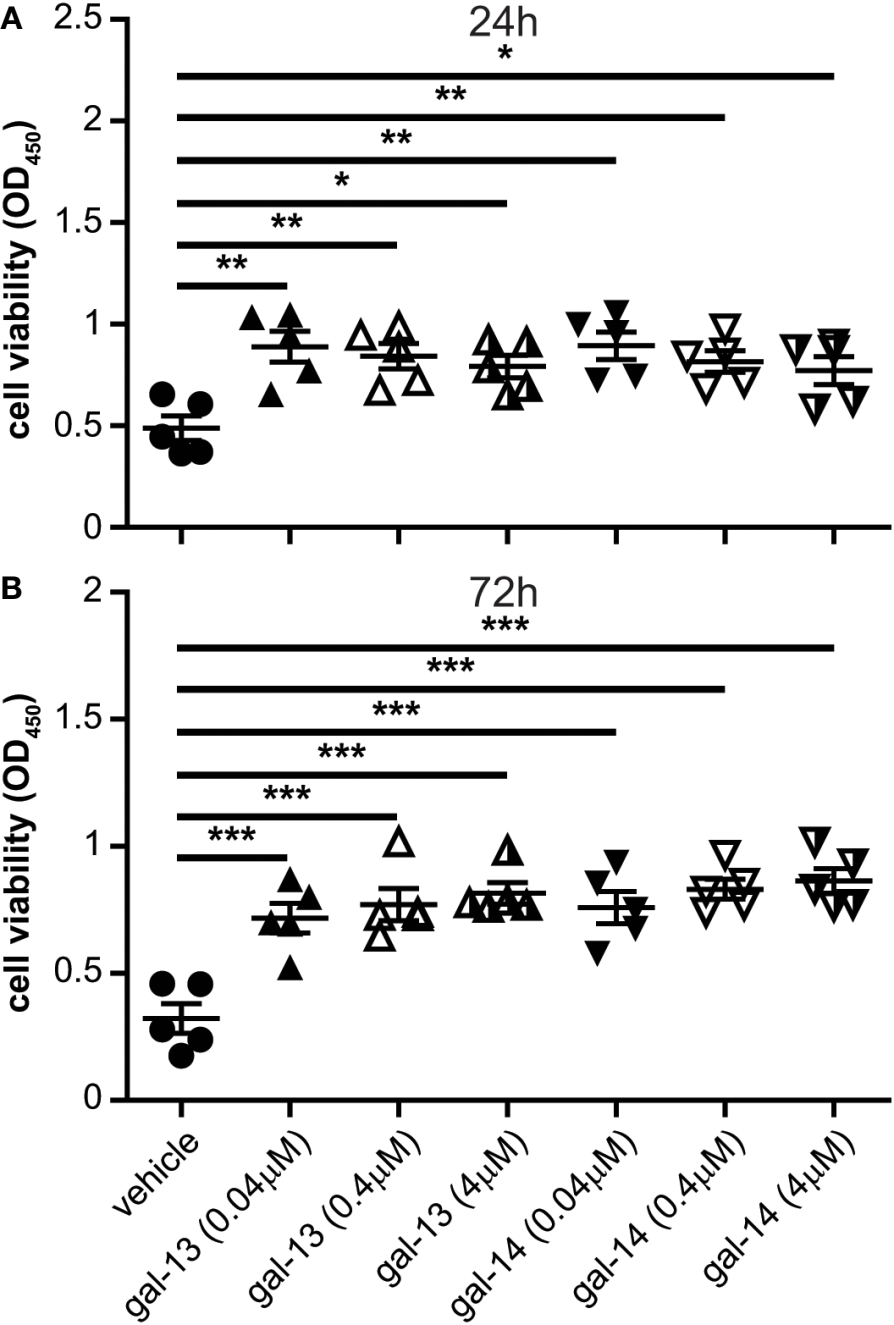
Gal-13 or gal-14 treatment increases the viability of immune cells. PBMCs were treated with gal-13, gal-14, or the vehicle for 24 and 72 hours. CCK-8 cell counting kit was used to examine cell viability. Graphs show the mean ± SEM of the optical density (450 nm) of viable cells after 24 hours **(A)** or 72 hours **(B)** of treatment (n=5). One-way ANOVA and Tukey *post-hoc* tests were used. Statistical significance was set at *p<0.05, **p<0.01, or ***p<0.001. PBMC: peripheral blood mononuclear cell, SEM: standard error of the mean.

Next, we examined the effect of recombinant gal-13 and gal-14 on the viability of PBMCs after 24 and 72 hours of treatment. Interestingly, gal-13 and gal-14 increased PBMC viability after 24 hours of treatment by 62–82% (gal-13) and 58–83% (gal-14), depending on the applied galectin concentration (**Figure 2A**). Ordered factor analysis revealed the significant concentration-dependent effect of both galectins (gal-13: p<0.01; gal-14: p<0.05).

After 72 hours of gal-13 and gal-14 administration, the viability of PBMCs showed even more of an increase than that of cells treated with the vehicle (gal-13: 123–153% increase; gal-14: 136–168% increase, depending on the galectin concentration) (**Figure 2B**). Herein, we also observed the significant concentration-dependent effect of both galectins (gal-13: p<10^-4^; gal-14: p<10^-5^).

**Figure 3:**
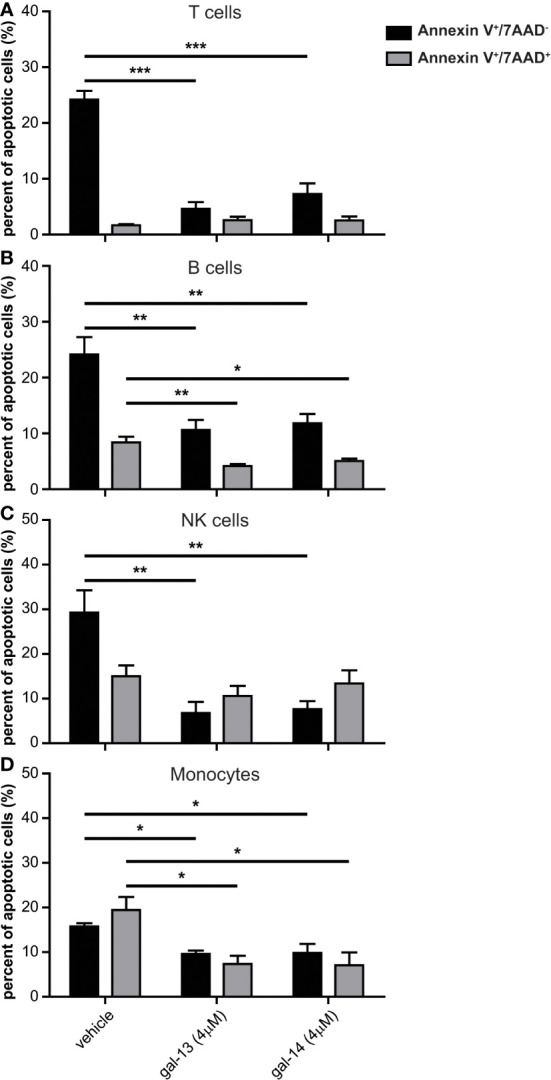
Gal-13 or gal-14 treatment reduces the apoptosis of PBMCs. PBMCs were incubated for 24 hours with gal-13, gal-14, or the vehicle. Graphs show the mean ± SEM of the percent of single positive (Annexin V +/7-AAD-, black bars) and double positive (Annexin V +/7-AAD +, grey bars) T lymphocytes, **(A)** B lymphocytes **(B)**, NK cells **(C)**, and monocytes **(D)** (n=4). One-way ANOVA and Tukey *post-hoc* tests were used. Statistical significance was set at *p<0.05, **p<0.01, or *** p<0.001. PBMC, peripheral blood mononuclear cell, SEM, standard error of the mean.

To examine whether the increased cell viability could be attributed to altered apoptosis or to proliferation, the impact of gal-13 and gal-14 on the apoptosis of immune cells was then explored (**Supplementary Figure 1** and **Figure 3**). In non-activated conditions, gal-13 and gal-14 reduced the proportion of early apoptotic T cells (gal-13: 19.6%; gal-14: 16.9%), B cells (gal-13: 13.6%; gal-14: 12.3%), NK cells (gal-13: 22.5%; gal-14: 21.7%), and monocytes (gal-13: 6.2%; gal-14: 5.9%) compared to vehicle-treated cells (**Figures 3A–D**). The proportion of late apoptotic B cells (**Figure 3B**) and monocytes (**Figure 3D**) was also reduced after gal-13 (by 4.2% and 12.1%, respectively) or gal-14 treatment (by 3.3% and 12.4%, respectively); however, the proportion of late apoptotic T cells and NK cells did not change upon treatment with placental galectins.

We also examined how gal-13 and gal-14 affect the proliferation of immune cell populations after 72 hours of treatment. Our results demonstrated that gal-13 and gal-14 had no impact on the proliferation of the PBMC populations under investigation (**Supplementary Figure 2**).

### Gal-13 and gal-14 treatment induce IL-8, IL-10, and IFN-γ production by immune cells

**Figure 4:**
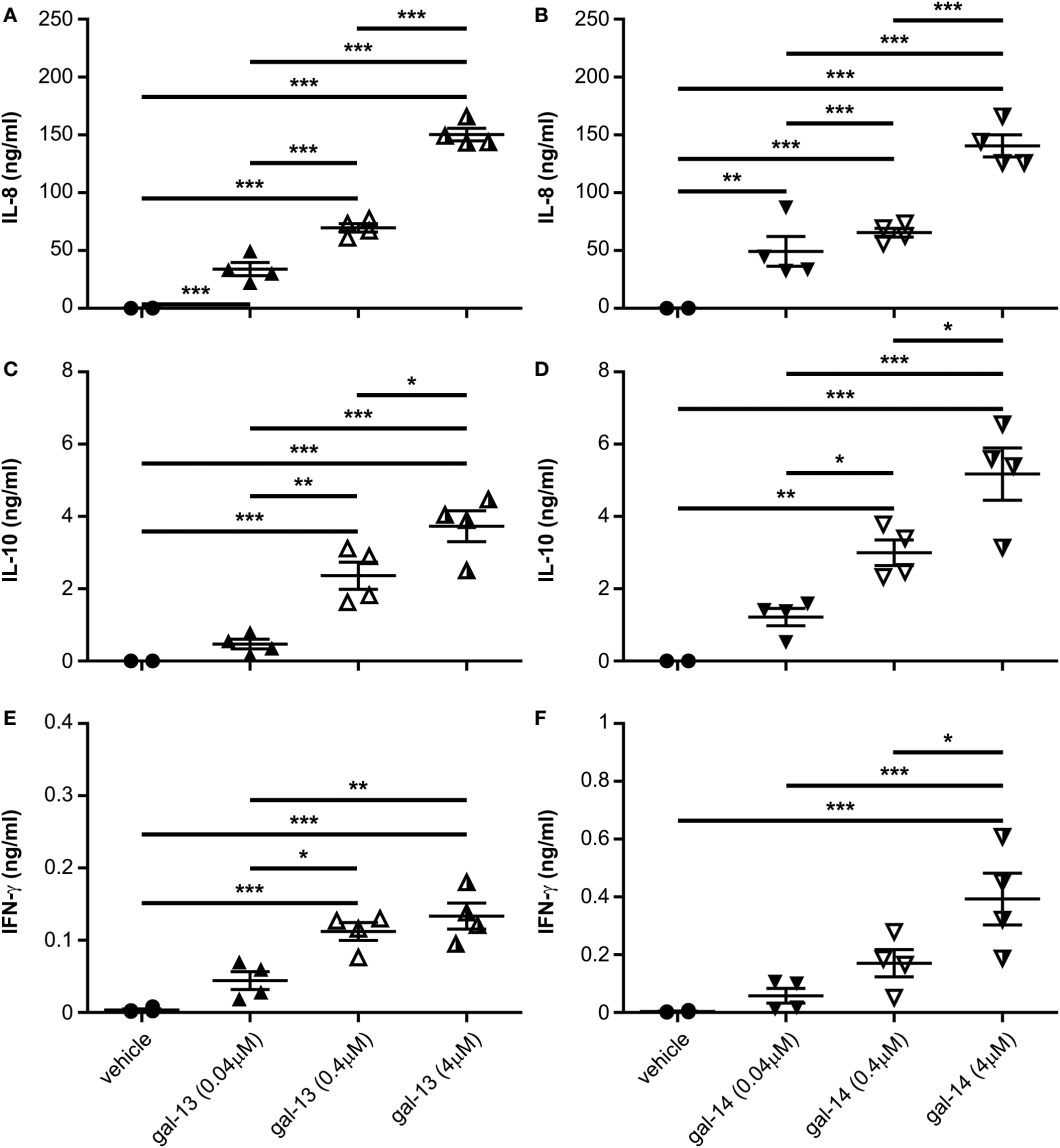
Gal-13 and gal-14 enhance the secretion of cytokines (IL-8, IL-10, IFN-γ) by immune cells. Cells were treated for 24 hours with different concentrations of gal-13 or gal-14. Cytokine production was measured by ELISA. Graphs show the mean ± SEM of IL-8 **(A, B)**, IL-10 **(C, D)**, and IFN-γ **(E, F)** cytokine concentrations (n=4). One-way ANOVA and Tukey *post-hoc* tests were used. Statistical significance was set at *p<0.05, **p<0.01, or ***p<0.001. IFN, interferon; IL, interleukin; SEM, standard error of the mean.

Based on our previous results concerning IL-8 production of T cells (78), we were interested in how placental galectins may regulate the production of an anti-inflammatory cytokine (IL-10) and a mainly pro-inflammatory cytokine (IFN-γ), both of which are important at the maternal-fetal interface (92–94). Therefore, PBMCs were treated with three different concentrations of gal-13 or gal-14 for 24 hours or for 72 hours. In comparison to the vehicle (mean: 0.137 ng/ml), IL-8 production increased after 24 hours of gal-13 or gal-14 treatment in a dose-dependent manner, ranging from 33.89 to 150.3 ng/ml (**Figures 4A, B**). Furthermore, a concentration-dependent effect of galectin treatment was also detected for IL-10 secretion into cell culture supernatants, ranging from 2.361 to 5.173 ng/ml (vehicle mean: 0.003 ng/ml) (**Figures 4C, D**). Twenty-four hours of gal-13 or gal-14 treatment increased IFN-γ secretion by immune cells as well (vehicle mean: 3.62 pg/ml, gal-13/gal-14 mean: from 112.1 to 392.5 pg/ml), although not as robustly as seen in IL-8 and IL-10 (**Figures 4E, F**). Ordered factor analysis revealed the significant concentration-dependent effect of both galectins in all comparisons (gal-13: IL-8: p<10^-5^; IL-10: p<10^-5^; IFN-γ: p<10^-5^; gal-14: IL-8: p<10^-5^; IL-10: p<10^-5^; IFN-γ: p<0.01). Similar outcomes, but with fewer significant values, were observed after the 72-hour galectin treatment (**Supplementary Figure 3**). Again, we observed significant concentration-dependent effect of both galectins in all comparisons (gal-13: IL-8: p<10^-5^; IL-10: p<10^-5^; IFN-γ: p<0.01; gal-14: IL-8: p<10^-5^; IL-10: p<10^-5^; IFN-γ: p<0.01). Overall, these results showed that treatment with either gal-13 or gal-14 increased the production of certain cytokines in immune cells to various extents—IL-8 production the most and IFN-γ production the least.

### Gal-13 and gal-14 cause phosphorylation of Erk1/2 and p38 MAPKs and of NF-ĸB in immune cells

**Figure 5:**
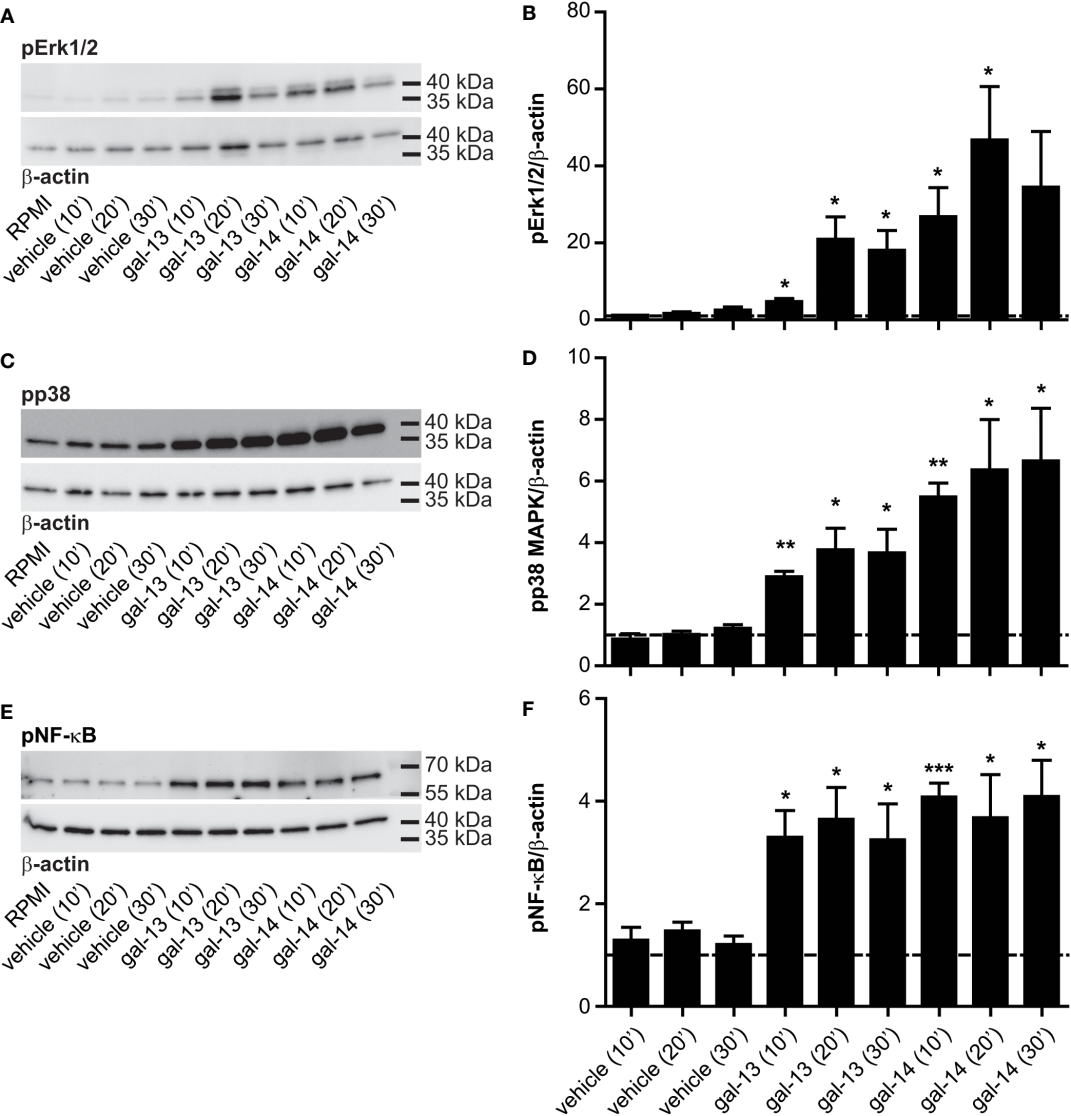
Galectin-13 and galectin-14 induce phosphorylation of Erk1/2, p38, and NF-κB in immune cells. The phosphorylation level of the mitogen-activated protein kinases Erk1/2 and p38, as well as transcription factor NF-κB was examined by Western blot. Freshly isolated PBMCs were incubated with the vehicle, gal-13, or gal-14 for 10, 20, and 30 minutes. Serum-free medium (RPMI-1640) was used as absolute control. Representative membrane strips for Erk1/2 **(A)**, p38 **(C)**, and NF-κB **(E)**, as well as for β-actin as a loading control. Graphs show the mean value ± SEM of densitometry results for Erk1/2 **(B)**, p38 **(D)**, and NF-κB **(F)** (n=4). One-sample t-test was used. Statistical significance was set at *p<0.05, **p<0.01, or ***p<0.001. Erk1/2, extracellular signal-regulated kinase 1/2; NF-κB, nuclear factor kappa B; SEM, standard error of the mean.

To identify signaling pathways upon gal-13 or gal-14 stimuli in PBMCs, the phosphorylation of two MAPKs (Erk1/2 and p38) and a transcription factor (NF-κB), all of which are crucial in immune cell functions, was examined. We found that 10–30 minutes of gal-13 or gal-14 treatment significantly phosphorylated Erk1/2 (gal-13: 4.40–13.85-fold; gal-14: 25.79–31.07-fold) (**Supplementary Figures 4A, B**, and **Figures 5A, B**) and p38 (gal-13: 3.05–3.77-fold; gal-14: 5.53–6.45-fold) (**Supplementary Figures 4C, D**, and **Figures 5C, D**). Furthermore, the phosphorylation of NF-κB was also increased after stimuli with recombinant galectins at all time points (gal-13: 2.47–2.69-fold; gal-14: 2.50–3.39-fold) (**Supplementary Figures 4E, F**, and **Figures 5E, F**).

## Discussion

### Principal findings of the study

The current study provides the following evidence: 1) Gal-13 and gal-14 bind to all investigated PBMC populations: the greatest extent to monocytes, followed by NK cells and B cells, and the least extent to T cells; 2) Gal-13 and gal-14 enhance the viability of PBMCs; 3) Gal-13 and gal-14 decrease the apoptosis of T cells, B cells, NK cells, and monocytes but have no effect on the proliferation of lymphocytic populations under the experimental conditions; 4) Gal-13 and gal-14 increase the production of cytokines in PBMCs, to the greatest extent in the pro-angiogenic and granulocyte chemoattractant IL-8, to a lesser extent in the main anti-inflammatory cytokine IL-10, and to the least extent in the pro-inflammatory and Th17 differentiation inhibiting IFN-γ production; and 5) Gal-13 and gal-14 induce Erk1/2 and p38 MAPKs and persistent NF-κB phosphorylation, which may contribute to the aforementioned effects by modulating the production of anti-apoptotic proteins and cytokines. These findings provide insights into the causal relationship between placenta-specific galectins and maternal-fetal immune regulation, which is further discussed below.

### Gal-13 and gal-14 increase cell viability *via* decreasing the apoptosis of immune cells

Several galectins affect immune responses by regulating cell viability, apoptosis, and proliferation, resulting in a shift in innate and adaptive immune responses at the maternal-fetal interfaces (30, 49, 50, 52). For instance, gal-9 is highly expressed by fetal cells (95) and suppresses cytotoxic T-cell functions by the induction of their apoptosis (96). In this study, we found that placental galectins increase the viability of the examined PBMC populations. Since viability assay alone could not determine whether reduced cell death or increased proliferation resulted in increased viability, we examined how gal-13 and gal-14 affected the apoptosis and proliferation of PBMCs. Both galectins reduced the early apoptosis of T and B lymphocytes, NK cells, and monocytes, and the late apoptosis of B cells and monocytes, in line with the results of the viability test. This is in accord with our recent study showing that gal-13 reduces the apoptosis of purified neutrophil granulocytes, thus promoting the polarization toward a placenta-growth-permissive phenotype, which contributes to trophoblast invasion and normal placentation during pregnancy (87). Our previous study found that gal-13 or gal-14 treatment increased the apoptosis of T cells (57, 60, 78). It is likely that the varying experimental conditions contributed to these differences since earlier studies (57, 60, 78) examined the effect of these galectins on the apoptosis of activated T lymphocytes or lymphocytes already kept in culture for several days, whereas, herein non-activated cells were used for the experiments after a short incubation period. These differences might have affected cell surface glycosylation events, which are known to significantly influence the overall effect of galectins on cell survival (97–99). Additionally, the effect of the microenvironment and surrounding immune cell populations in the mixed cell culture may have also impacted the overall anti-apoptotic activity of gal-13 and gal-14.

An important difference might be that apoptosis-inducing or -inhibiting effects of galectins are highly influenced by the cell-surface glycosylation status of target cells (98). This is tightly regulated by their activation status, e.g., resting, activated, and various differentiation states, determined by the local microenvironment and the set of stimuli reaching the cell. For example, activated Th1 and Th17 cells express a repertoire of glycans required for gal-1 binding and are susceptible to gal-1-induced apoptosis. For example, it was demonstrated that activated T cells and Th1 cells express a repertoire of glycans required for gal-1 binding, which makes them susceptible to gal-1-induced apoptosis, whereas Th2 cells are less responsive to gal-1 binding and gal-1-induced apoptosis due to their 2,6-sialylation on the cell-surface glycoproteins (99). Activated T cells, Th1 cells, and Th2 cells, however, displayed comparable levels of gal-3-induced apoptosis, proving that Th2 cells’ resistance to gal-1-induced death is specific and does not signify a generally higher resistance to apoptosis (99). Another study by Kopcow et al. found that peripheral and decidual T cells have different glycophenotypes, thus a distinct gal-1-binding capacity compatible with higher sensitivity to gal-1 of decidual T cells (40). This finding is supported by the substantial proportion of apoptotic T cells in the decidua due to T-cell death mediated by local gal-1 (48). As neither gal-13 nor gal-14 had an effect on the proliferation of T and B lymphocytes as well as NK cells, we concluded that the anti-apoptotic activity of these placental galectins is responsible for the observed increase in PBMC viability.

### Gal-13 and gal-14 induce the production of IL-8, IL-10, and IFN-γ cytokines in immune cells

Earlier, gal-13 was described to increase the production of IL-1ɑ and IL-6 in PBMCs (86) as well as gal-13 and gal-14 to enhance IL-8 production in T cells (78). Herein, we were interested in determining whether gal-13 and gal-14 may influence the production of other cytokines in PBMCs as well. Our experimental conditions differed from the study applied by Kliman et al. (86), also by using DTT and higher galectin concentrations, which might have led to the observation of galectin-induced production of IL-8, IL-10, and IFN-γ in PBMCs in our study. IL-8 can contribute to the development of blood supply at the maternal-fetal interface through its pro-angiogenic effect on endothelial cells (100, 101). IL-8 is also a chemoattractant for neutrophils, so by attracting these cells to the maternal-fetal interface and promoting their phenotypic change, gal-13 and gal-14 may contribute to placentation and the formation of a proper immune environment (87). The increased production of IL-10, which can be produced by several immune cells, may contribute to the shift toward a tolerogenic immune environment in pregnancy (94). Previous publications described that gal-9 increased the production of IL-4, IL-6, and IL-10 by PBMCs (102) and that gal-1 also increased the production of IL-10 but decreased the production of IFN-γ by T lymphocytes (103). Since IFN-γ is produced by NK cells, inhibits Th17 differentiation (104), and promotes decidual NK cell differentiation and placental development (93, 105), a modest IFN-γ production upon gal-13 and gal-14 treatment may also show profound effects at the maternal-fetal interface. Decidual T cells express a high level of IFN-γ besides IL-4 (106), which may also relate to local effects of progesterone (107). Therefore, in the future, it would be interesting to study whether placental galectins can contribute to the phenotypic changes of these immune cells in the decidua.

### Gal-13 and gal-14 regulate immune cells by the activation of Erk1/2, p38, and NF-κB signaling pathways

Galectins were found to influence immune cell functions *via* key signaling pathways, e.g., the MAPK and NF-κB pathways (108–113). However, the interaction partners and receptors of gal-13 and gal-14 expressed on immune cells, and the effects of these galectins on signal transduction, have not yet been revealed. Herein, we showed that gal-13 and gal-14 treatment induced the phosphorylation of Erk1/2 and p38 MAPKs and the persistent phosphorylation of the p65 subunit of the NF-κB transcription factor. These signaling molecules are essential for the generation of immune responses, such as inflammation, pathogen recognition, T- and B-cell responses, and lymphocyte longevity (15, 114–117), and several galectins, e.g., gal-1, gal-8, gal-9, have been shown to induce or inhibit their activation (108, 110, 118, 119). For example, it was found that gal-8 induced apoptosis in Jurkat T cells by triggering an Erk1/2-dependent death pathway (108). In another study, human monocytes upregulated FcγRI and downregulated MHC-II surface expression in an Erk1/2-dependent manner upon gal-1 treatment (110). It was also demonstrated that gal-9 increased the phosphorylation of Akt, c-Jun N-terminal kinase, and Erk1/2—the key kinases involved in NK cell function—in peripheral TIM-3^+^ NK cells, which led to their suppressed activation (118). Furthermore, gal-14 was found to regulate cell function through the interaction with the transcription factor c-Rel, a subunit of NF-ĸB, which has many functions in pathways related to inflammation, apoptosis, cell growth, and cell differentiation, among others (119). However, in the current study, gal-14 was overexpressed in a HeLa (cervical cancer) cell line, in which gal-14 is normally not expressed, so the results may not be physiologically relevant (119). In light of these findings, it will be valuable to investigate additional signaling pathways that may be regulated by gal-13 and gal-14.

Of significance, while intracellular galectins can bind to proteins, extracellular galectins bind to glycan sidechains of glycoproteins at cell surfaces. Therefore, various glycoproteins with the same carbohydrate moieties can be considered as receptors for placental galectins, similar to other galectins. Indeed, we have shown previously that gal-13 differentially bound to red blood cells due to its varying affinity for blood-group AB0 glycans. For example, gal-13 binds blood-group AB erythrocytes with the strongest while blood-group B erythrocytes with the weakest affinity (120). The affinity of gal-13 and gal-14 to certain mono- or disaccharide ligands was also revealed (53, 59, 62). It is an interesting question whether placental galectins can bind to the glycan chains on the surface of extracellular vesicles secreted or deported from the placenta, which would add another layer of signaling events between the fetus and the mother. Earlier studies showed that gal-13 is indeed localized in/on extracellular vesicles, but the ways of this binding require further examination (62, 65). Future studies also need to investigate the cell surface glycoprotein receptors of these galectins on white blood cells and how the glycosylation pattern of these cells may determine the strength of galectin binding and galectin-induced signaling events.

### Strengths and limitations of the study

Strengths: 1) A large set of functional assays was applied to examine the effects of gal-13 and gal-14 on immune cells obtained from non-pregnant female donors; 2) we used a mixed PBMC population instead of purified cell populations to better mimic the physiological conditions in human circulation. However, future investigations of isolated immune cell populations shall reveal more insights into the effects of gal-13 and gal-14 on specific immune cell types; and 3) recombinant galectins were expressed in a modified bacterial vector to avoid lipopolysaccharide contamination and biased immune cell activation. Limitations: 1) The applied quantities of gal-13 and gal-14 were supraphysiologic relative to that found in human circulation. However, these concentrations were similar to those previously used by us and others (10 to 100 µg/mL) to study the functional effects of a large set of galectins (121, 122). Furthermore, galectin blood concentrations do not represent their effective tissue or cell surface concentrations, where galectin-glycan lattices present a high local galectin concentration (123–126). Another technical rationale for using larger galectin concentrations was to prevent subunit dissociation in the DTT-containing solvent, which could lead to functional changes (127); 2) due to the use of mixed PBMC populations, we cannot exclude the effects of immune cell populations on each other; and 3) immune cells were isolated from non-pregnant female donors, which do not represent a perfect match for those cells that could be isolated from pregnant women. However, this method was the only option to avoid pre-exposure of immune cells to gal-13/gal-14 binding, which could have biased our studies. Nevertheless, future studies shall also characterize the effects of gal-13 and gal-14 on peripheral blood and decidual leukocytes isolated from pregnant women, since pregnancy hormones may strongly impact the glycosylation pattern and the galectin-binding capacity of these cells.

## Conclusions

**Figure 6:**
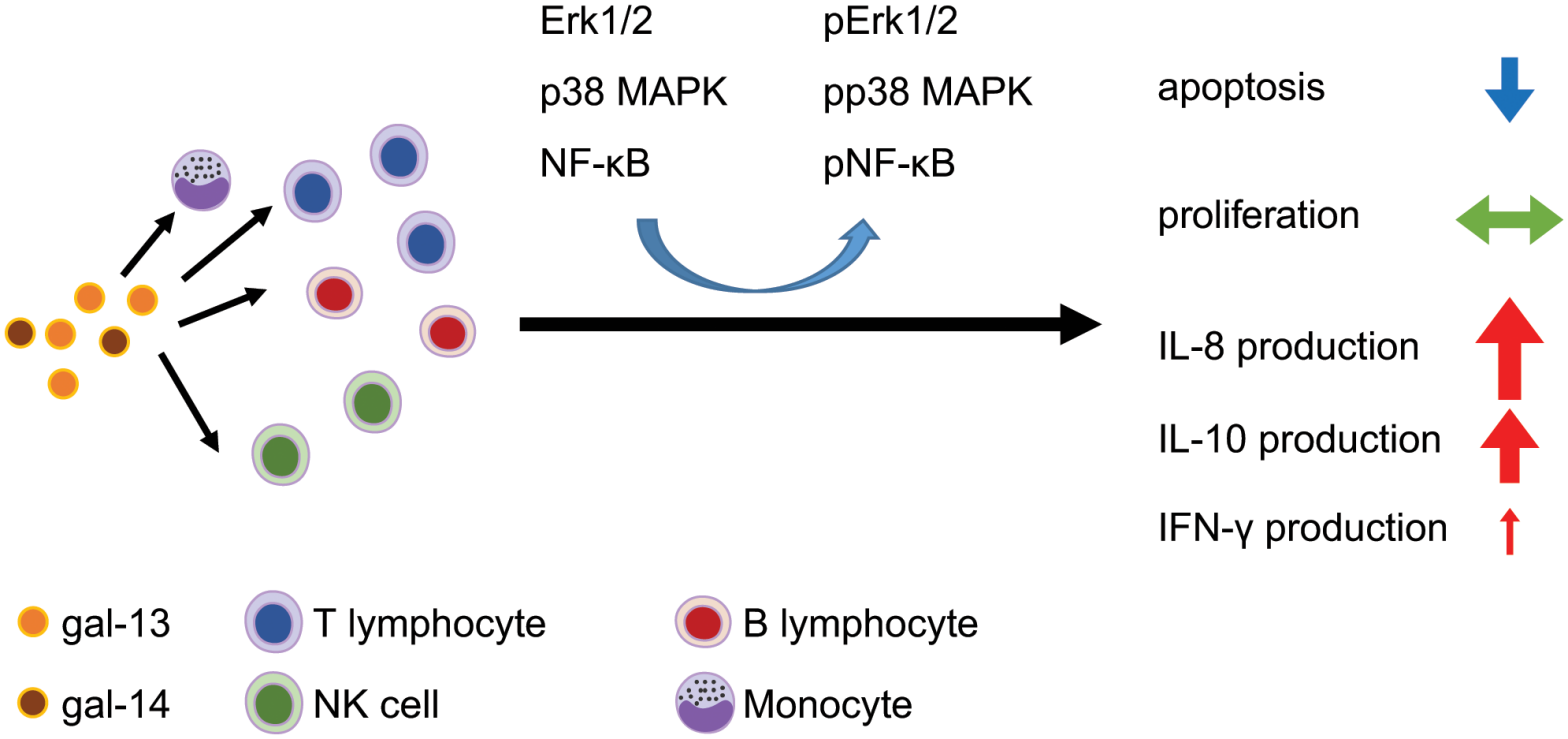
The effects of placental galectins on leukocyte functions. Gal-13 and gal-14 bind to the surface of immune cell populations where they activate Erk1/2, p38 MAPK, and NF-κB signaling pathways. These signaling effects of placenta-specific galectins increase the viability of leukocytes, decrease the apoptosis of immune cells without influencing the proliferation of cells, and induce the production of IL-8, IL-10, and IFN-γ in a different manner. Erk1/2, extracellular signal-regulated kinase 1/2; IFN, interferon; IL, interleukin; MAPK, mitogen-activated protein kinase; NF-κB, nuclear factor kappa B.

We demonstrate herein that gal-13 and gal-14 bind to non-activated PBMC populations, increase their viability by reducing their apoptosis, and induce the production of IL-8, IL-10, and IFN-γ, probably by the involvement of Erk1/2, p38, and NF-ĸB signaling pathways (**Figure 6**). Our findings suggest that these placental galectins regulate immune cells and their decreased expression in obstetrical syndromes may play a role in the severe immunopathology of these syndromes.

## Data availability statement

The raw data supporting the conclusions of this article willbe made available by the authors, without undue reservation.

## Ethics statement

Ethical review and approval were not required for the study on human participants in accordance with the local legislation and institutional requirements. Written informed consent for participation was not required for this study in accordance with the national legislation and the institutional requirements.

## Author contributions

OO, JM, NGT, and AB conceptualized the study and designed research. OO, ET, JK, and AB performed research. RR, NGT, and AB contributed new reagents/analytic tools/clinical specimens. OO, RR, JK, MP, DG, SR, AT, OE, ZP, JM, NGT, and AB analyzed and interpreted data. All authors contributed to the article and approved the submitted version.

## References

[1] Szekeres-Bartho J Wegmann TG. A progesterone-dependent immunomodulatory protein alters the Th1/Th2 balance. J Reprod Immunol (1996) 31(1-2):81–95. doi: 10.1016/0165-0378(96)00964-3 8887124

[2] Abrahams VM Kim YM Straszewski SL Romero R Mor G. Macrophages and apoptotic cell clearance during pregnancy. Am J Reprod Immunol (2004) 51(4):275–82. doi: 10.1111/j.1600-0897.2004.00156.x 15212680

[3] Mor G Romero R Aldo PB Abrahams VM. Is the trophoblast an immune regulator? the role of toll-like receptors during pregnancy. Crit Rev Immunol (2005) 25(5):375–88. doi: 10.1615/CritRevImmunol.v25.i5.30 16167887

[4] Richani K Soto E Romero R Espinoza J Chaiworapongsa T Nien JK. Normal pregnancy is characterized by systemic activation of the complement system. J Matern Fetal Neonatal Med (2005) 17(4):239–45. doi: 10.1080/14767050500072722 PMC1421513 16147832

[5] Blois SM Kammerer U Alba Soto C Tometten MC Shaikly V Barrientos G. Dendritic cells: key to fetal tolerance? Biol Reprod (2007) 77(4):590–8. doi: 10.1095/biolreprod.107.060632 17596562

[6] Kalkunte S Chichester CO Gotsch F Sentman CL Romero R Sharma S. Evolution of non-cytotoxic uterine natural killer cells. Am J Reprod Immunol (2008) 59(5):425–32. doi: 10.1111/j.1600-0897.2008.00595.x PMC3042548 18405313

[7] Mor G. Inflammation and pregnancy: the role of toll-like receptors in trophoblast-immune interaction. Ann N Y Acad Sci (2008) 1127:121–8. doi: 10.1196/annals.1434.006 18443339

[8] Gomez-Lopez N Vega-Sanchez R Castillo-Castrejon M Romero R Cubeiro-Arreola K Vadillo-Ortega F. Evidence for a role for the adaptive immune response in human term parturition. Am J Reprod Immunol (2013) 69(3):212–30. doi: 10.1111/aji.12074 PMC3600361 23347265

[9] El-Azzamy H Balogh A Romero R Xu Y LaJeunesse C Plazyo O. Characteristic changes in decidual gene expression signature in spontaneous term parturition. J Pathol Transl Med (2017) 51(3):264–83. doi: 10.4132/jptm.2016.12.20 PMC5445200 28226203

[10] Szekeres-Bartho J Sucurovic S Mulac-Jericevic B. The role of extracellular vesicles and PIBF in embryo-maternal immune-interactions. Front Immunol (2018) 9:2890. doi: 10.3389/fimmu.2018.02890 30619262 PMC6300489

[11] Vacca P Vitale C Munari E Cassatella MA Mingari MC Moretta L. Human innate lymphoid cells: Their functional and cellular interactions in decidua. Front Immunol (2018) 9:1897. doi: 10.3389/fimmu.2018.01897 30154799 PMC6102343

[12] Ander SE Diamond MS Coyne CB. Immune responses at the maternal-fetal interface. Sci Immunol (2019) 4(31):eaat6114. doi: 10.1126/sciimmunol.aat6114 30635356 PMC6744611

[13] Mor G Cardenas I Abrahams V Guller S. Inflammation and pregnancy: the role of the immune system at the implantation site. Ann N Y Acad Sci (2011) 1221:80–7. doi: 10.1111/j.1749-6632.2010.05938.x PMC3078586 21401634

[14] Racicot K Kwon JY Aldo P Silasi M Mor G. Understanding the complexity of the immune system during pregnancy. Am J Reprod Immunol (2014) 72(2):107–16. doi: 10.1111/aji.12289 PMC6800182 24995526

[15] Aghaeepour N Ganio EA McIlwain D Tsai AS Tingle M Van Gassen S. An immune clock of human pregnancy. Sci Immunol (2017) 2(15):eaan2946. doi: 10.1126/sciimmunol.aan2946 28864494 PMC5701281

[16] Peterson LS Stelzer IA Tsai AS Ghaemi MS Han X Ando K. Multiomic immune clockworks of pregnancy. Semin Immunopathol (2020) 42(4):397–412. doi: 10.1007/s00281-019-00772-1 32020337 PMC7508753

[17] Piccinni MP Robertson SA Saito S. Editorial: Adaptive immunity in pregnancy. Front Immunol (2021) 12:770242. doi: 10.3389/fimmu.2021.770242 34671364 PMC8520997

[18] Dam TK Brewer CF. Lectins as pattern recognition molecules: the effects of epitope density in innate immunity. Glycobiology (2010) 20(3):270–9. doi: 10.1093/glycob/cwp186 19939826

[19] Miller D Motomura K Galaz J Gershater M Lee ED Romero R. Cellular immune responses in the pathophysiology of preeclampsia. J Leukoc Biol (2022) 111(1):237–60. doi: 10.1002/JLB.5RU1120-787RR PMC8511357 33847419

[20] Than NG Romero R Balogh A Karpati E Mastrolia SA Staretz-Chacham O. Galectins: Double-edged swords in the cross-roads of pregnancy complications and female reproductive tract inflammation and neoplasia. J Pathol Transl Med (2015) 49(3):181–208. doi: 10.4132/jptm.2015.02.25 26018511 PMC4440931

[21] Vasta GR Ahmed H Nita-Lazar M Banerjee A Pasek M Shridhar S. Galectins as self/non-self recognition receptors in innate and adaptive immunity: an unresolved paradox. Front Immunol (2012) 3:199. doi: 10.3389/fimmu.2012.00199 22811679 PMC3396283

[22] Toldi G Svec P Vasarhelyi B Meszaros G Rigo J Tulassay T. Decreased number of FoxP3+ regulatory T cells in preeclampsia. Acta Obstet Gynecol Scand (2008) 87(11):1229–33. doi: 10.1080/00016340802389470 19016357

[23] Hahn S Hasler P Vokalova L van Breda SV Lapaire O Than NG. The role of neutrophil activation in determining the outcome of pregnancy and modulation by hormones and/or cytokines. Clin Exp Immunol (2019) 198(1):24–36. doi: 10.1111/cei.13278 30768780 PMC6718280

[24] Murrieta-Coxca JM Fuentes-Zacarias P Ospina-Prieto S Markert UR Morales-Prieto DM. Synergies of extracellular vesicles and microchimerism in promoting immunotolerance during pregnancy. Front Immunol (2022) 13:837281. doi: 10.3389/fimmu.2022.837281 35844513 PMC9285877

[25] Depierreux DM Kieckbusch J Shreeve N Hawkes DA Marsh B Blelloch R. Beyond maternal tolerance: Education of uterine natural killer cells by maternal MHC drives fetal growth. Front Immunol (2022) 13:808227. doi: 10.3389/fimmu.2022.808227 35619712 PMC9127083

[26] Zhuang B Shang J Yao Y. HLA-G: An important mediator of maternal-fetal immune-tolerance. Front Immunol (2021) 12:744324. doi: 10.3389/fimmu.2021.744324 34777357 PMC8586502

[27] Ding J Zhang Y Cai X Diao L Yang C Yang J. Crosstalk between trophoblast and macrophage at the maternal-fetal interface: Current status and future perspectives. Front Immunol (2021) 12:758281. doi: 10.3389/fimmu.2021.758281 34745133 PMC8566971

[28] Xu L Li Y Sang Y Li DJ Du M. Crosstalk between trophoblasts and decidual immune cells: The cornerstone of maternal-fetal immunotolerance. Front Immunol (2021) 12:642392. doi: 10.3389/fimmu.2021.642392 33717198 PMC7947923

[29] Sharma S Banerjee S Krueger PM Blois SM. Immunobiology of gestational diabetes mellitus in post-medawar era. Front Immunol (2021) 12:758267. doi: 10.3389/fimmu.2021.758267 35046934 PMC8761800

[30] Molvarec A Blois SM Stenczer B Toldi G Tirado-Gonzalez I Ito M. Peripheral blood galectin-1-expressing T and natural killer cells in normal pregnancy and preeclampsia. Clin Immunol (2011) 139(1):48–56. doi: 10.1016/j.clim.2010.12.018 21292557

[31] Meggyes M Miko E Polgar B Bogar B Farkas B Illes Z. Peripheral blood TIM-3 positive NK and CD8+ T cells throughout pregnancy: TIM-3/galectin-9 interaction and its possible role during pregnancy. PloS One (2014) 9(3):e92371. doi: 10.1371/journal.pone.0092371 24651720 PMC3961322

[32] Miko E Meggyes M Bogar B Schmitz N Barakonyi A Varnagy A. Involvement of galectin-9/TIM-3 pathway in the systemic inflammatory response in early-onset preeclampsia. PloS One (2013) 8(8):e71811. doi: 10.1371/journal.pone.0071811 23936526 PMC3732281

[33] Blois SM Barrientos G. Galectin signature in normal pregnancy and preeclampsia. J Reprod Immunol (2014) 101-102:127–34. doi: 10.1016/j.jri.2013.05.005 23953090

[34] Barrientos G Freitag N Tirado-Gonzalez I Unverdorben L Jeschke U Thijssen VL. Involvement of galectin-1 in reproduction: past, present and future. Hum Reprod Update (2014) 20(2):175–93. doi: 10.1093/humupd/dmt040 24077937

[35] Blois SM Verlohren S Wu G Clark G Dell A Haslam SM. Role of galectin-glycan circuits in reproduction: from healthy pregnancy to preterm birth (PTB). Semin Immunopathol (2020) 42(4):469–86. doi: 10.1007/s00281-020-00801-4 PMC7508936 32601855

[36] Blois SM Dveksler G Vasta GR Freitag N Blanchard V Barrientos G. Pregnancy galectinology: Insights into a complex network of glycan binding proteins. Front Immunol (2019) 10:1166. doi: 10.3389/fimmu.2019.01166 31231368 PMC6558399

[37] Tirado-Gonzalez I Freitag N Barrientos G Shaikly V Nagaeva O Strand M. Galectin-1 influences trophoblast immune evasion and emerges as a predictive factor for the outcome of pregnancy. Mol Hum Reprod (2013) 19(1):43–53. doi: 10.1093/molehr/gas043 23002109

[38] Blois SM Ilarregui JM Tometten M Garcia M Orsal AS Cordo-Russo R. A pivotal role for galectin-1 in fetomaternal tolerance. Nat Med (2007) 13(12):1450–7. doi: 10.1038/nm1680 18026113

[39] Meister S Hahn L Beyer S Mannewitz M Perleberg C Schnell K. Regulatory T cell apoptosis during preeclampsia may be prevented by gal-2. Int J Mol Sci (2022) 23(3):1880. doi: 10.3390/ijms23031880 PMC8836599 35163802

[40] Blidner AG Rabinovich GA. 'Sweetening' pregnancy: galectins at the fetomaternal interface. Am J Reprod Immunol (2013) 69(4):369–82. doi: 10.1111/aji.12090 23406009

[41] Ramhorst RE Giribaldi L Fraccaroli L Toscano MA Stupirski JC Romero MD. Galectin-1 confers immune privilege to human trophoblast: implications in recurrent fetal loss. Glycobiology (2012) 22(10):1374–86. doi: 10.1093/glycob/cws104 22752006

[42] Menkhorst E Than NG Jeschke U Barrientos G Szereday L Dveksler G. Medawar's PostEra: Galectins emerged as key players during fetal-maternal glycoimmune adaptation. Front Immunol (2021) 12:784473. doi: 10.3389/fimmu.2021.784473 34975875 PMC8715898

[43] Freitag N Tirado-Gonzalez I Barrientos G Herse F Thijssen VL Weedon-Fekjaer SM. Interfering with gal-1-mediated angiogenesis contributes to the pathogenesis of preeclampsia. Proc Natl Acad Sci U S A (2013) 110(28):11451–6. doi: 10.1073/pnas.1303707110 PMC3710834 23798433

[44] Cooper DN. Galectinomics: finding themes in complexity. Biochim Biophys Acta (2002) 1572(2-3):209–31. doi: 10.1016/S0304-4165(02)00310-0 12223271

[45] Gitt MA Barondes SH. Evidence that a human soluble beta-galactoside-binding lectin is encoded by a family of genes. Proc Natl Acad Sci U S A (1986) 83(20):7603–7. doi: 10.1073/pnas.83.20.7603 PMC386769 3020551

[46] Than NG Romero R Kim CJ McGowen MR Papp Z Wildman DE. Galectins: guardians of eutherian pregnancy at the maternal-fetal interface. Trends Endocrinol Metab (2012) 23(1):23–31. doi: 10.1016/j.tem.2011.09.003 22036528 PMC3640805

[47] Miko E Meggyes M Doba K Barakonyi A Szereday L. Immune checkpoint molecules in reproductive immunology. Front Immunol (2019) 10:846. doi: 10.3389/fimmu.2019.00846 31057559 PMC6482223

[48] Kopcow HD Rosetti F Leung Y Allan DS Kutok JL Strominger JL. T Cell apoptosis at the maternal-fetal interface in early human pregnancy, involvement of galectin-1. Proc Natl Acad Sci U S A (2008) 105(47):18472–7. doi: 10.1073/pnas.0809233105 PMC2587580 19011096

[49] Than NG Romero R Erez O Weckle A Tarca AL Hotra J. Emergence of hormonal and redox regulation of galectin-1 in placental mammals: implication in maternal-fetal immune tolerance. Proc Natl Acad Sci U S A (2008) 105(41):15819–24. doi: 10.1073/pnas.0807606105 PMC2556362 18824694

[50] Terness P Kallikourdis M Betz AG Rabinovich GA Saito S Clark DA. Tolerance signaling molecules and pregnancy: IDO, galectins, and the renaissance of regulatory T cells. Am J Reprod Immunol (2007) 58(3):238–54. doi: 10.1111/j.1600-0897.2007.00510.x 17681041

[51] Sun J Yang M Ban Y Gao W Song B Wang Y. Tim-3 is upregulated in NK cells during early pregnancy and inhibits NK cytotoxicity toward trophoblast in galectin-9 dependent pathway. PloS One (2016) 11(1):e0147186. doi: 10.1371/journal.pone.0147186 26789128 PMC4720443

[52] Hu XH Tang MX Mor G Liao AH. Tim-3: Expression on immune cells and roles at the maternal-fetal interface. J Reprod Immunol (2016) 118:92–9. doi: 10.1016/j.jri.2016.10.113 27792886

[53] Than NG Pick E Bellyei S Szigeti A Burger O Berente Z. Functional analyses of placental protein 13/galectin-13. Eur J Biochem (2004) 271(6):1065–78. doi: 10.1111/j.1432-1033.2004.04004.x 15009185

[54] Vicovac L Jankovic M Cuperlovic M. Galectin-1 and -3 in cells of the first trimester placental bed. Hum Reprod (1998) 13(3):730–5. doi: 10.1093/humrep/13.3.730 9572443

[55] Jeschke U Mayr D Schiessl B Mylonas I Schulze S Kuhn C. Expression of galectin-1, -3 (gal-1, gal-3) and the thomsen-friedenreich (TF) antigen in normal, IUGR, preeclamptic and HELLP placentas. Placenta (2007) 28(11-12):1165–73. doi: 10.1016/j.placenta.2007.06.006 17664004

[56] Van den Brule FA Fernandez PL Buicu C Liu FT Jackers P Lambotte R. Differential expression of galectin-1 and galectin-3 during first trimester human embryogenesis. Dev Dyn (1997) 209(4):399–405. doi: 10.1002/(SICI)1097-0177(199708)209:4<399::AID-AJA7>3.0.CO;2-D 9264263

[57] Than NG Balogh A Romero R Karpati E Erez O Szilagyi A. Placental protein 13 (PP13) - a placental immunoregulatory galectin protecting pregnancy. Front Immunol (2014) 5:348. doi: 10.3389/fimmu.2014.00348 25191322 PMC4138504

[58] Maquoi E van den Brule FA Castronovo V Foidart JM. Changes in the distribution pattern of galectin-1 and galectin-3 in human placenta correlates with the differentiation pathways of trophoblasts. Placenta (1997) 18(5-6):433–9. doi: 10.1016/S0143-4004(97)80044-6 9250706

[59] Than NG Romero R Goodman M Weckle A Xing J Dong Z. A primate subfamily of galectins expressed at the maternal-fetal interface that promote immune cell death. Proc Natl Acad Sci U S A (2009) 106(24):9731–6. doi: 10.1073/pnas.0903568106 PMC2689813 19497882

[60] Than NG Romero R Xu Y Erez O Xu Z Bhatti G. Evolutionary origins of the placental expression of chromosome 19 cluster galectins and their complex dysregulation in preeclampsia. Placenta (2014) 35(11):855–65. doi: 10.1016/j.placenta.2014.07.015 PMC4203431 25266889

[61] Than NG Sumegi B Than GN Berente Z Bohn H. Isolation and sequence analysis of a cDNA encoding human placental tissue protein 13 (PP13), a new lysophospholipase, homologue of human eosinophil charcot-Leyden crystal protein. Placenta (1999) 20(8):703–10. doi: 10.1053/plac.1999.0436 10527825

[62] Than NG Abdul Rahman O Magenheim R Nagy B Fule T Hargitai B. Placental protein 13 (galectin-13) has decreased placental expression but increased shedding and maternal serum concentrations in patients presenting with preterm pre-eclampsia and HELLP syndrome. Virchows Arch (2008) 453(4):387–400. doi: 10.1007/s00428-008-0658-x 18791734 PMC2775473

[63] Huppertz B Sammar M Chefetz I Neumaier-Wagner P Bartz C Meiri H. Longitudinal determination of serum placental protein 13 during development of preeclampsia. Fetal Diagn Ther (2008) 24(3):230–6. doi: 10.1159/000151344 18753763

[64] Balogh A Pozsgay J Matko J Dong Z Kim CJ Varkonyi T. Placental protein 13 (PP13/galectin-13) undergoes lipid raft-associated subcellular redistribution in the syncytiotrophoblast in preterm preeclampsia and HELLP syndrome. Am J Obstet Gynecol (2011) 205(2):156 e1–14. doi: 10.1016/j.ajog.2011.03.023 PMC3527099 21596368

[65] Sammar M Dragovic R Meiri H Vatish M Sharabi-Nov A Sargent I. Reduced placental protein 13 (PP13) in placental derived syncytiotrophoblast extracellular vesicles in preeclampsia - a novel tool to study the impaired cargo transmission of the placenta to the maternal organs. Placenta (2018) 66:17–25. doi: 10.1016/j.placenta.2018.04.013 29884298

[66] Madar-Shapiro L Karady I Trahtenherts A Syngelaki A Akolekar R Poon L. Predicting the risk to develop preeclampsia in the first trimester combining promoter variant -98A/C of LGALS13 (Placental protein 13), black ethnicity, previous preeclampsia, obesity, and maternal age. Fetal Diagn Ther (2018) 43(4):250–65. doi: 10.1159/000477933 PMC5882584 28728156

[67] Romero R Kusanovic JP Than NG Erez O Gotsch F Espinoza J. First-trimester maternal serum PP13 in the risk assessment for preeclampsia. Am J Obstet Gynecol (2008) 199(2):122 e1– e11. doi: 10.1016/j.ajog.2008.01.013 PMC2784814 18539259

[68] Chafetz I Kuhnreich I Sammar M Tal Y Gibor Y Meiri H. First-trimester placental protein 13 screening for preeclampsia and intrauterine growth restriction. Am J Obstet Gynecol (2007) 197(1):35 e1–7. doi: 10.1016/j.ajog.2007.02.025 17618748

[69] Meiri H Sammar M Herzog A Grimpel YI Fihaman G Cohen A. Prediction of preeclampsia by placental protein 13 and background risk factors and its prevention by aspirin. J Perinat Med (2014) 42(5):591–601. doi: 10.1515/jpm-2013-0298 24607918

[70] Wortelboer EJ Koster MP Cuckle HS Stoutenbeek PH Schielen PC Visser GH. First-trimester placental protein 13 and placental growth factor: markers for identification of women destined to develop early-onset pre-eclampsia. BJOG (2010) 117(11):1384–9. doi: 10.1111/j.1471-0528.2010.02690.x 20840693

[71] Saito S Shiozaki A Nakashima A Sakai M Sasaki Y. The role of the immune system in preeclampsia. Mol Aspects Med (2007) 28(2):192–209. doi: 10.1016/j.mam.2007.02.006 17433431

[72] Perez-Sepulveda A Torres MJ Khoury M Illanes SE. Innate immune system and preeclampsia. Front Immunol (2014) 5:244. doi: 10.3389/fimmu.2014.00244 24904591 PMC4033071

[73] Geldenhuys J Rossouw TM Lombaard HA Ehlers MM Kock MM. Disruption in the regulation of immune responses in the placental subtype of preeclampsia. Front Immunol (2018) 9:1659. doi: 10.3389/fimmu.2018.01659 30079067 PMC6062603

[74] Laresgoiti-Servitje E. A leading role for the immune system in the pathophysiology of preeclampsia. J Leukoc Biol (2013) 94(2):247–57. doi: 10.1189/jlb.1112603 23633414

[75] Than NG Posta M Gyorffy D Orosz L Orosz G Rossi SW. Early pathways, biomarkers, and four distinct molecular subclasses of preeclampsia: The intersection of clinical, pathological, and high-dimensional biology studies. Placenta (2022) 125:10–9. doi: 10.1016/j.placenta.2022.03.009 PMC9261837 35428514

[76] Eikmans M Morales-Prieto DM van der Hoorn ML Markert UR. Editorial: Immunological challenges around pregnancy complications associated with failures of maternal tolerance to the fetus. Front Immunol (2022) 13:983739. doi: 10.3389/fimmu.2022.983739 35979367 PMC9376944

[77] Than NG Romero R Tarca AL Kekesi KA Xu Y Xu Z. Integrated systems biology approach identifies novel maternal and placental pathways of preeclampsia. Front Immunol (2018) 9:1661. doi: 10.3389/fimmu.2018.01661 30135684 PMC6092567

[78] Balogh A Toth E Romero R Parej K Csala D Szenasi NL. Placental galectins are key players in regulating the maternal adaptive immune response. Front Immunol (2019) 10:1240. doi: 10.3389/fimmu.2019.01240 31275299 PMC6593412

[79] Wu C Thalhamer T Franca RF Xiao S Wang C Hotta C. Galectin-9-CD44 interaction enhances stability and function of adaptive regulatory T cells. Immunity (2014) 41(2):270–82. doi: 10.1016/j.immuni.2014.06.011 PMC4219323 25065622

[80] Sano H Hsu DK Apgar JR Yu L Sharma BB Kuwabara I. Critical role of galectin-3 in phagocytosis by macrophages. J Clin Invest (2003) 112(3):389–97. doi: 10.1172/JCI200317592 PMC166291 12897206

[81] Sanchez-Fueyo A Tian J Picarella D Domenig C Zheng XX Sabatos CA. Tim-3 inhibits T helper type 1-mediated auto- and alloimmune responses and promotes immunological tolerance. Nat Immunol (2003) 4(11):1093–101. doi: 10.1038/ni987 14556005

[82] Chen HY Liu FT Yang RY. Roles of galectin-3 in immune responses. Arch Immunol Ther Exp (Warsz) (2005) 53(6):497–504. 16407782

[83] Garin MI Chu CC Golshayan D Cernuda-Morollon E Wait R Lechler RI. Galectin-1: a key effector of regulation mediated by CD4+CD25+ T cells. Blood (2007) 109(5):2058–65. doi: 10.1182/blood-2006-04-016451 17110462

[84] Oomizu S Arikawa T Niki T Kadowaki T Ueno M Nishi N. Cell surface galectin-9 expressing Th cells regulate Th17 and Foxp3+ treg development by galectin-9 secretion. PloS One (2012) 7(11):e48574. doi: 10.1371/journal.pone.0048574 23144904 PMC3492452

[85] Oomizu S Arikawa T Niki T Kadowaki T Ueno M Nishi N. Galectin-9 suppresses Th17 cell development in an IL-2-dependent but Tim-3-independent manner. Clin Immunol (2012) 143(1):51–8. doi: 10.1016/j.clim.2012.01.004 22341088

[86] Kliman HJ Sammar M Grimpel YI Lynch SK Milano KM Pick E. Placental protein 13 and decidual zones of necrosis: an immunologic diversion that may be linked to preeclampsia. Reprod Sci (2012) 19(1):16–30. doi: 10.1177/1933719111424445 21989657

[87] Vokalova L Balogh A Toth E Van Breda SV Schafer G Hoesli I. Placental protein 13 (Galectin-13) polarizes neutrophils toward an immune regulatory phenotype. Front Immunol (2020) 11:145. doi: 10.3389/fimmu.2020.00145 32117288 PMC7028707

[88] Than NG Erez O Wildman DE Tarca AL Edwin SS Abbas A. Severe preeclampsia is characterized by increased placental expression of galectin-1. J Matern Fetal Neonatal Med (2008) 21(7):429–42. doi: 10.1080/14767050802041961 PMC2775462 18570123

[89] Hirashima C Ohkuchi A Nagayama S Suzuki H Takahashi K Ogoyama M. Galectin-1 as a novel risk factor for both gestational hypertension and preeclampsia, specifially its expression at a low level in the second trimester and a high level after onset. Hypertens Res (2018) 41(1):45–52. doi: 10.1038/hr.2017.85 28978981

[90] Pankiewicz K Szczerba E Fijalkowska A Szamotulska K Szewczyk G Issat T. The association between serum galectin-3 level and its placental production in patients with preeclampsia. J Physiol Pharmacol (2020) 71(6):845–56. doi: 10.26402/jpp.2020.6.08 33727431

[91] Freitag N Tirado-Gonzalez I Barrientos G Powell KL Boehm-Sturm P Koch SP. Galectin-3 deficiency in pregnancy increases the risk of fetal growth restriction (FGR) *via* placental insufficiency. Cell Death Dis (2020) 11(7):560. doi: 10.1038/s41419-020-02791-5 32703931 PMC7378206

[92] Yang F Zheng Q Jin L. Dynamic function and composition changes of immune cells during normal and pathological pregnancy at the maternal-fetal interface. Front Immunol (2019) 10:2317. doi: 10.3389/fimmu.2019.02317 31681264 PMC6813251

[93] Murphy SP Tayade C Ashkar AA Hatta K Zhang J Croy BA. Interferon gamma in successful pregnancies. Biol Reprod (2009) 80(5):848–59. doi: 10.1095/biolreprod.108.073353 PMC2849832 19164174

[94] Cheng SB Sharma S. Interleukin-10: a pleiotropic regulator in pregnancy. Am J Reprod Immunol (2015) 73(6):487–500. doi: 10.1111/aji.12329 25269386 PMC4382460

[95] Selno ATH Schlichtner S Yasinska IM Sakhnevych SS Fiedler W Wellbrock J. Transforming growth factor beta type 1 (TGF-beta) and hypoxia-inducible factor 1 (HIF-1) transcription complex as master regulators of the immunosuppressive protein galectin-9 expression in human cancer and embryonic cells. Aging (Albany NY) (2020) 12(23):23478–96. doi: 10.18632/aging.202343 PMC7762483 33295886

[96] Schlichtner S Meyer NH Yasinska IM Aliu N Berger SM Gibbs BF. Functional role of galectin-9 in directing human innate immune reactions to gram-negative bacteria and T cell apoptosis. Int Immunopharmacol (2021) 100:108155. doi: 10.1016/j.intimp.2021.108155 34543981

[97] Pereira MS Alves I Vicente M Campar A Silva MC Padrao NA. Glycans as key checkpoints of T cell activity and function. Front Immunol (2018) 9:2754. doi: 10.3389/fimmu.2018.02754 30538706 PMC6277680

[98] Daniels MA Hogquist KA Jameson SC. Sweet 'n' sour: the impact of differential glycosylation on T cell responses. Nat Immunol (2002) 3(10):903–10. doi: 10.1038/ni1002-903 12352967

[99] Toscano MA Bianco GA Ilarregui JM Croci DO Correale J Hernandez JD. Differential glycosylation of TH1, TH2 and TH-17 effector cells selectively regulates susceptibility to cell death. Nat Immunol (2007) 8(8):825–34. doi: 10.1038/ni1482 17589510

[100] Li A Dubey S Varney ML Dave BJ Singh RK. IL-8 directly enhanced endothelial cell survival, proliferation, and matrix metalloproteinases production and regulated angiogenesis. J Immunol (2003) 170(6):3369–76. doi: 10.4049/jimmunol.170.6.3369 12626597

[101] Lash GE Ernerudh J. Decidual cytokines and pregnancy complications: focus on spontaneous miscarriage. J Reprod Immunol (2015) 108:83–9. doi: 10.1016/j.jri.2015.02.003 25771398

[102] Enninga EA Nevala WK Holtan SG Leontovich AA Markovic SN. Galectin-9 modulates immunity by promoting Th2/M2 differentiation and impacts survival in patients with metastatic melanoma. Melanoma Res (2016) 26(5):429–41. doi: 10.1097/CMR.0000000000000281 PMC5929986 27455380

[103] van der Leij J van den Berg A Blokzijl T Harms G van Goor H Zwiers P. Dimeric galectin-1 induces IL-10 production in T-lymphocytes: an important tool in the regulation of the immune response. J Pathol (2004) 204(5):511–8. doi: 10.1002/path.1671 15538736

[104] Chong WP van Panhuys N Chen J Silver PB Jittayasothorn Y Mattapallil MJ. NK-DC crosstalk controls the autopathogenic Th17 response through an innate IFN-gamma-IL-27 axis. J Exp Med (2015) 212(10):1739–52. doi: 10.1084/jem.20141678 PMC4577839 26347474

[105] Zhou JZ Way SS Chen K. Immunology of the uterine and vaginal mucosae. Trends Immunol (2018) 39(4):302–14. doi: 10.1016/j.it.2018.01.007 29433961

[106] Powell RM Lissauer D Tamblyn J Beggs A Cox P Moss P. Decidual T cells exhibit a highly differentiated phenotype and demonstrate potential fetal specificity and a strong transcriptional response to IFN. J Immunol (2017) 199(10):3406–17. doi: 10.4049/jimmunol.1700114 PMC5679367 28986438

[107] Lissauer D Eldershaw SA Inman CF Coomarasamy A Moss PA Kilby MD. Progesterone promotes maternal-fetal tolerance by reducing human maternal T-cell polyfunctionality and inducing a specific cytokine profile. Eur J Immunol (2015) 45(10):2858–72. doi: 10.1002/eji.201445404 PMC4833190 26249148

[108] Norambuena A Metz C Vicuna L Silva A Pardo E Oyanadel C. Galectin-8 induces apoptosis in jurkat T cells by phosphatidic acid-mediated ERK1/2 activation supported by protein kinase a down-regulation. J Biol Chem (2009) 284(19):12670–9. doi: 10.1074/jbc.M808949200 PMC2675996 19276072

[109] Hsu DK Chen HY Liu FT. Galectin-3 regulates T-cell functions. Immunol Rev (2009) 230(1):114–27. doi: 10.1111/j.1600-065X.2009.00798.x 19594632

[110] Barrionuevo P Beigier-Bompadre M Ilarregui JM Toscano MA Bianco GA Isturiz MA. A novel function for galectin-1 at the crossroad of innate and adaptive immunity: galectin-1 regulates monocyte/macrophage physiology through a nonapoptotic ERK-dependent pathway. J Immunol (2007) 178(1):436–45. doi: 10.4049/jimmunol.178.1.436 17182582

[111] Dai SY Nakagawa R Itoh A Murakami H Kashio Y Abe H. Galectin-9 induces maturation of human monocyte-derived dendritic cells. J Immunol (2005) 175(5):2974–81. doi: 10.4049/jimmunol.175.5.2974 16116184

[112] Hong SH Shin JS Chung H Park CG. Galectin-4 interaction with CD14 triggers the differentiation of monocytes into macrophage-like cells *via* the MAPK signaling pathway. Immune Netw (2019) 19(3):e17. doi: 10.4110/in.2019.19.e17 31281714 PMC6597441

[113] Walzel H Blach M Hirabayashi J Arata Y Kasai K Brock J. Galectin-induced activation of the transcription factors NFAT and AP-1 in human jurkat T-lymphocytes. Cell Signal (2002) 14(10):861–8. doi: 10.1016/S0898-6568(02)00035-9 12135707

[114] Hayden MS Ghosh S. NF-kappaB in immunobiology. Cell Res (2011) 21(2):223–44. doi: 10.1038/cr.2011.13 PMC3193440 21243012

[115] Gomez-Chavez F Correa D Navarrete-Meneses P Cancino-Diaz JC Cancino-Diaz ME Rodriguez-Martinez S. NF-kappaB and its regulators during pregnancy. Front Immunol (2021) 12:679106. doi: 10.3389/fimmu.2021.679106 34025678 PMC8131829

[116] Ariyakumar G Morris JM McKelvey KJ Ashton AW McCracken SA. NF-kappaB regulation in maternal immunity during normal and IUGR pregnancies. Sci Rep (2021) 11(1):20971. doi: 10.1038/s41598-021-00430-3 34697371 PMC8545974

[117] Fragiadakis GK Baca QJ Gherardini PF Ganio EA Gaudilliere DK Tingle M. Mapping the fetomaternal peripheral immune system at term pregnancy. J Immunol (2016) 197(11):4482–92. doi: 10.4049/jimmunol.1601195 PMC5125527 27793998

[118] Li Y Zhang J Zhang D Hong X Tao Y Wang S. Tim-3 signaling in peripheral NK cells promotes maternal-fetal immune tolerance and alleviates pregnancy loss. Sci Signal (2017) 10(498):eaah4323. doi: 10.1126/scisignal.aah4323 28951537

[119] Si Y Li Y Yang T Li X Ayala GJ Mayo KH. Structure-function studies of galectin-14, an important effector molecule in embryology. FEBS J (2021) 288(3):1041–55. doi: 10.1111/febs.15441 32525264

[120] Than NG Romero R Meiri H Erez O Xu Y Tarquini F. PP13, maternal ABO blood groups and the risk assessment of pregnancy complications. PloS One (2011) 6(7):e21564. doi: 10.1371/journal.pone.0021564 21799738 PMC3143125

[121] Yildirim C Vogel DY Hollander MR Baggen JM Fontijn RD Nieuwenhuis S. Galectin-2 induces a proinflammatory, anti-arteriogenic phenotype in monocytes and macrophages. PloS One (2015) 10(4):e0124347. doi: 10.1371/journal.pone.0124347 25884209 PMC4401781

[122] Stowell SR Karmakar S Stowell CJ Dias-Baruffi M McEver RP Cummings RD. Human galectin-1, -2, and -4 induce surface exposure of phosphatidylserine in activated human neutrophils but not in activated T cells. Blood (2007) 109(1):219–27. doi: 10.1182/blood-2006-03-007153 PMC1785076 16940423

[123] Crocker PR. Mammalian carbohydrate recognition systems. Results and Problems in Cell Differentiation [Internet] (2001). doi: 10.1007/978-3-540-46410-5

[124] Nabi IR Shankar J Dennis JW. The galectin lattice at a glance. J Cell Sci (2015) 128(13):2213–9. doi: 10.1242/jcs.151159 26092931

[125] Bonzi J Bornet O Betzi S Kasper BT Mahal LK Mancini SJ. Pre-b cell receptor binding to galectin-1 modifies galectin-1/carbohydrate affinity to modulate specific galectin-1/glycan lattice interactions. Nat Commun (2015) 6:6194. doi: 10.1038/ncomms7194 25708191

[126] Rabinovich GA Toscano MA. Turning 'sweet' on immunity: galectin-glycan interactions in immune tolerance and inflammation. Nat Rev Immunol (2009) 9(5):338–52. doi: 10.1038/nri2536 19365409

[127] Cedeno-Laurent F Dimitroff CJ. Galectin-1 research in T cell immunity: past, present and future. Clin Immunol (2012) 142(2):107–16. doi: 10.1016/j.clim.2011.09.011 PMC3266984 22019770

