# Supplementary material for: Placental galectins regulate innate and adaptive immune responses in pregnancy
Source: Front Immunol. 2022 Dec 28;13:1088024. doi: 10.3389/fimmu.2022.1088024 (PMC9832025; doi:10.3389/fimmu.2022.1088024)
Supplement: Supplementary file 1 [file DataSheet_1.docx]

**Supplementary material**

**Supplementary table 1. Flow cytometry antibodies/reagents**

| Antibody/reagent | Clonality | Amount/test | Source (Cat. #) |
| --- | --- | --- | --- |
| human FcR blocking reagent | - | 20 µl from 1: 10 dilution | Miltenyi Biotec  (130-059-901) |
| Gal-13-CF488 recombinant protein | monoclonal | 5 µg | MBP-tag modified version of the gal-13 expression plasmid provided by Prof. R. Romero (PRB, NIH) |
| Gal-14-CF488 recombinant protein | monoclonal | 5 µg | MBP-tag modified version of the gal-14 expression plasmid provided by Prof. R. Romero (PRB, NIH) |
| anti-human CD3-APC | monoclonal | 2.5 µl | BioLegend (317318) |
| anti-human CD14-APC | monoclonal | 2.5 µl | BioLegend (301808) |
| anti-human CD19-PerCP | monoclonal | 2.5 µl | BioLegend (302227) |
| anti-human CD20-FITC | monoclonal | 2.5 µl | BioLegend (302304) |
| anti-human CD56-APC/Fire 750 | monoclonal | 2.5 µl | BioLegend (362553) |
| anti-human CD56-APC/eF780 | monoclonal | 2.5 µl | Thermo Fisher Scientific  (47-0567-42) |
| Zombie Violet | - | 100 µl from 1:500 dilution | BioLegend (423113) |
| annexin V-PE | - | 2.5 µl | BioLegend (640908) |
| 7-AAD | - | 2.5 µl | BioLegend (640922) |

**Supplementaty table 2. Western blot conditions**

| Primary antibody  (dilution) | Primary antibody source  (Cat. #) | HRP-antibody  (dilution) | HRP-antibody source (Cat. #) |
| --- | --- | --- | --- |
| Anti-phospho-Erk1/2  (1:2,000) | Cell Signaling, monoclonal rabbit IgG (#4370) | Goat-anti-rabbit IgG (1:10,000) | Novex (A24537) |
| Anti-phospho-p38 MAPK  (1:2,000) | Cell Signaling, monoclonal rabbit IgG (#4511) | Goat-anti-rabbit IgG (1:10,000) | Novex (A24537) |
| Anti-phopspho-NF-κB  (1:1,000) | Cell Signaling, monoclonal rabbit IgG (#3033) | Goat-anti-rabbit IgG (1:10,000) | Novex (A24537) |
| Anti-β-actin  (1:2,500) | Abcam, monoclonal mouse IgG (#MA5-15739) | Goat-anti-mouse IgG (1:2,500) | Novex (A16072) |


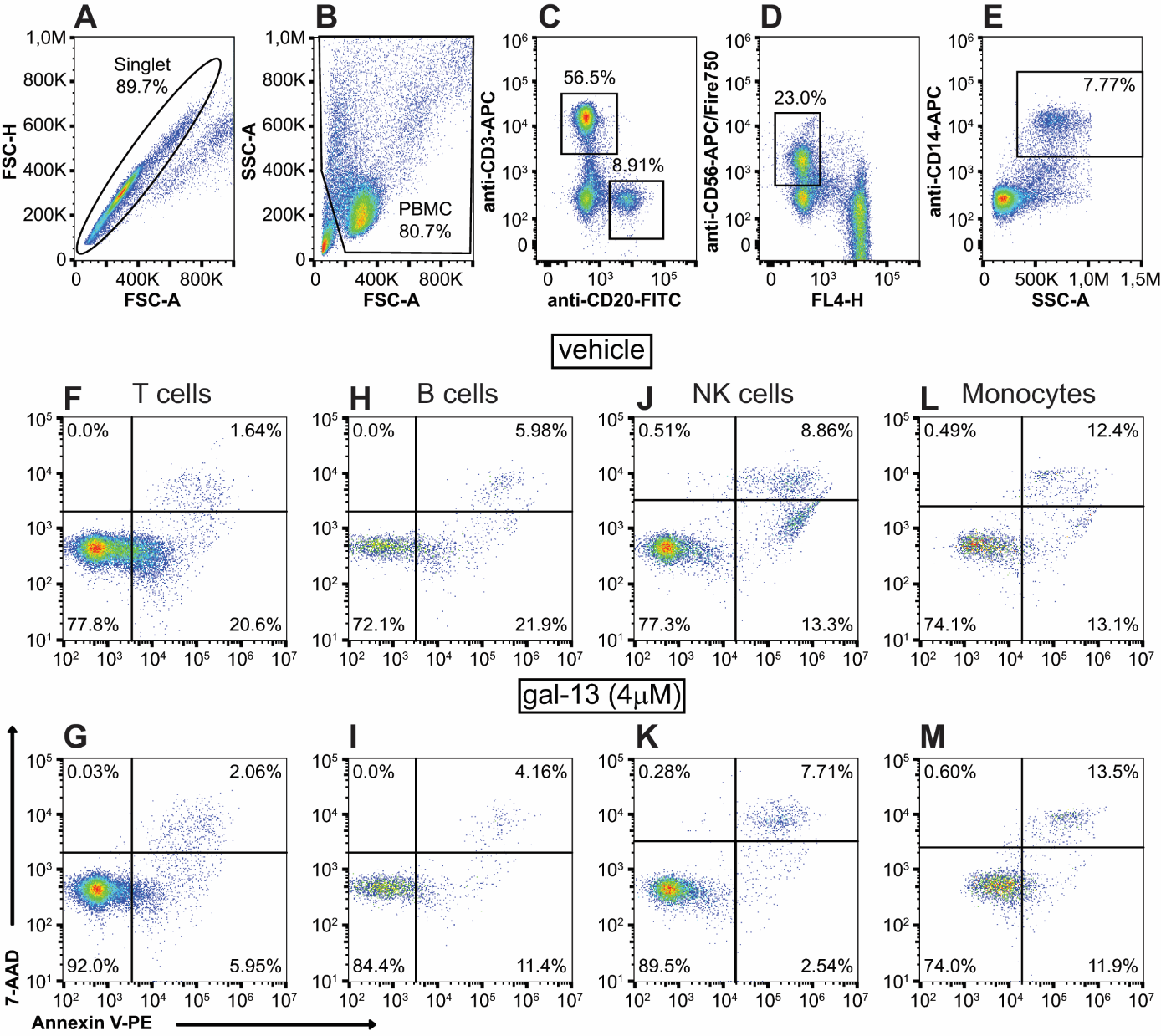


**Supplementary Figure 1. Effect of placental galectin treatment on apoptosis of immune cells.** Peripheral blood mononuclear cells were treated with gal-13 for 24 hours. To detect apoptosis in each PBMC population, cells were labeled with antibodies specific for CD3, CD20, CD56, and CD14 cell surface markers, as well as with annexin V and 7-AAD reagents. First, FSC-A/FSC-H dot plot (**A**) was used to gate on PBMC singlets (individual cells). The FSC-A/SSC-A dot plot (**B**) was used to gate on the PBMC populations. In the next step, anti-CD3-APC and anti-CD20-FITC (**C**), anti-CD56-APC/F750 (**D**), as well as anti-CD14-APC (**E**) antibodies were used to gate on T cells, B cells, NK cells, and monocytes, respectively. Representative dot plots show annexin V-PE and 7-AAD staining of vehicle- or gal-13-treated T lymphocytes (**F, G**), B lymphocytes (**H, I**), NK cells (**J, K**), and monocytes (**L, M**) (n=4). PBMCs: peripheral blood mononuclear cells.


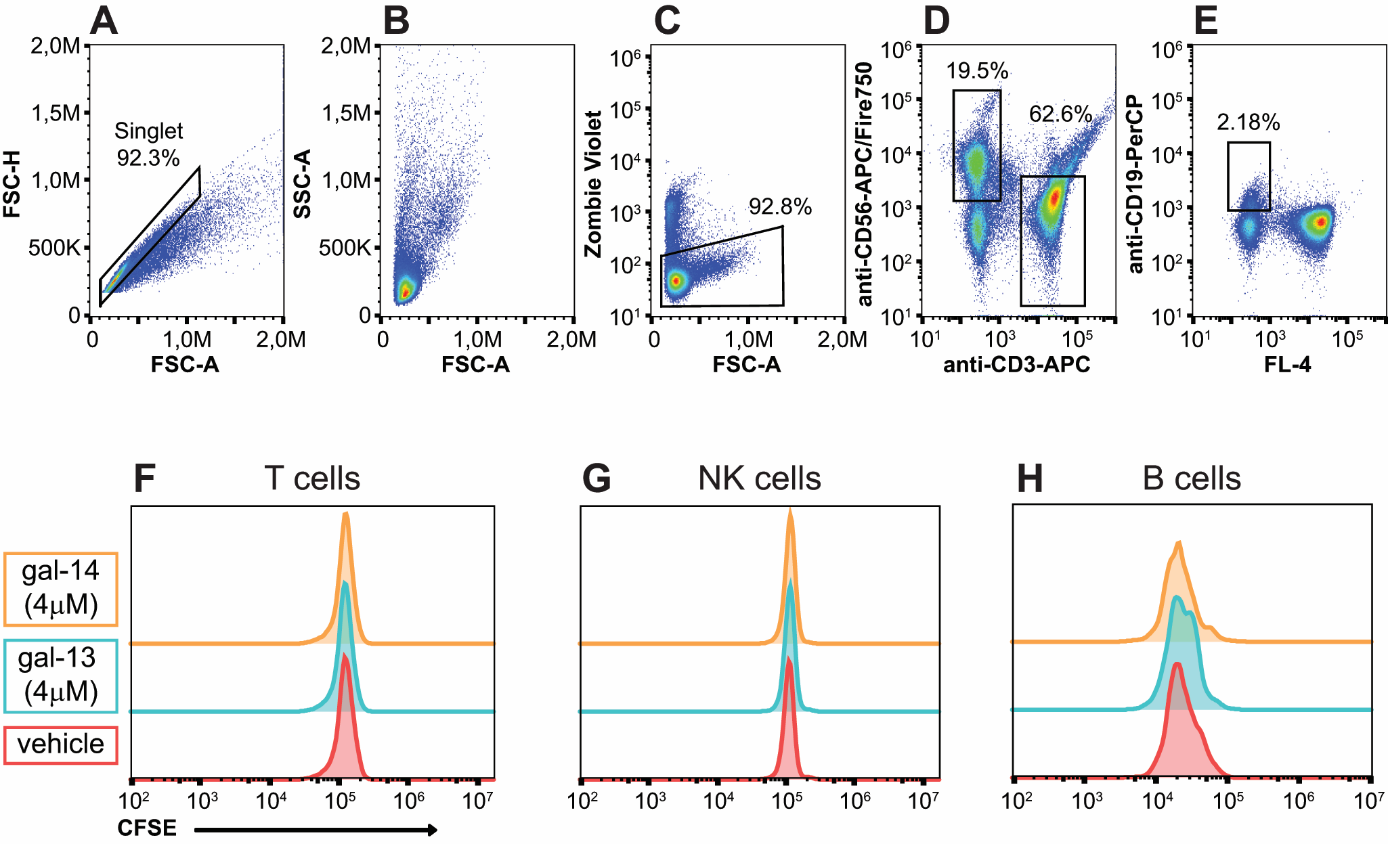


**Supplementary Figure 2. Effect of placental galectin treatment on proliferation of immune cells.** CFSE-labeled PBMCs were treated with gal-13 or gal-14 for 72 hours. Dot plots show the gating strategy. First, FSC-A/FSC-H dot plot (**A**) was used to gate on PBMC singlets (individual cells). FSC-A/PB450-H (Zombie Violet dye) dot plot (**B**) was used to gate on viable cells. The FSC-A/SSC-A dot plot (**C**) was checked to ensure that previous gating well delineated the PBMC population. In the next step, anti-CD3-APC and anti-CD56-APC/eF780 (**D**), as well as anti-CD19-PerCP (**E**) antibodies were used to gate on T cells, NK cells, and B cells, respectively. Representative histograms show CFSE-staining of vehicle- (red), gal-13- (blue), or gal-14-treated (orange) T-, NK-, and B cells (**F, G, and H,** respectively) (n=4). CFSE: carboxyfluorescein succinimidyl ester, PBMCs: peripheral blood mononuclear cells.


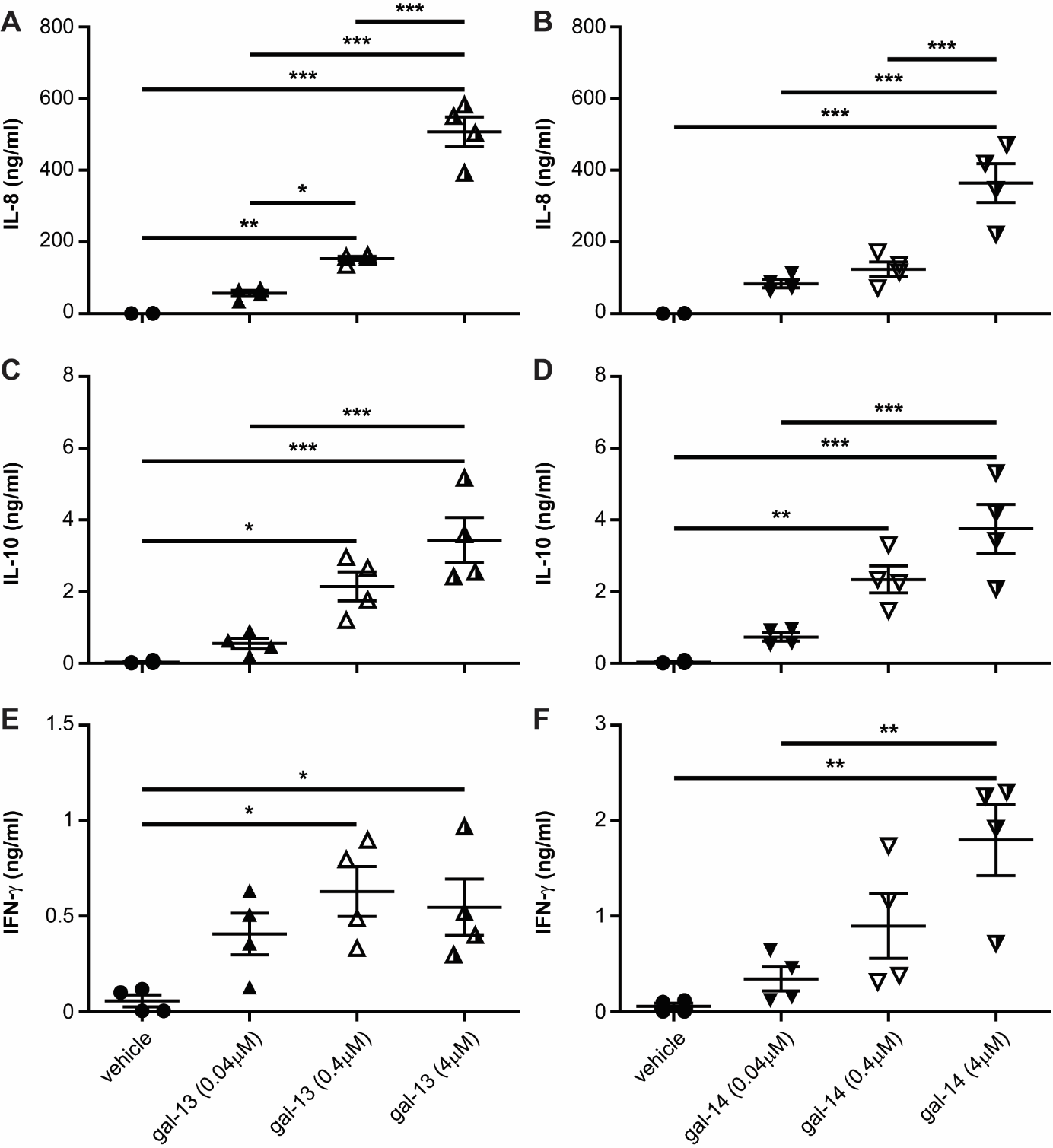


**Supplementary Figure 3. Galectin-13 or galectin-14 enhances the secretion of cytokines by immune cells.** Cells were treated for 72 hours with different concentrations of gal-13 or gal-14. Cytokine production was measured by ELISA. Graphs show the mean ± SEM of IL-8 (**A, B**), IL-10 (**C, D**), IFN-γ (**E, F**) cytokine concentrations (n=4), using one-way ANOVA and Tukey post-hoc test (* p <0.05, ** p <0.01, * ** p <0.001). SEM: standard error of the mean.


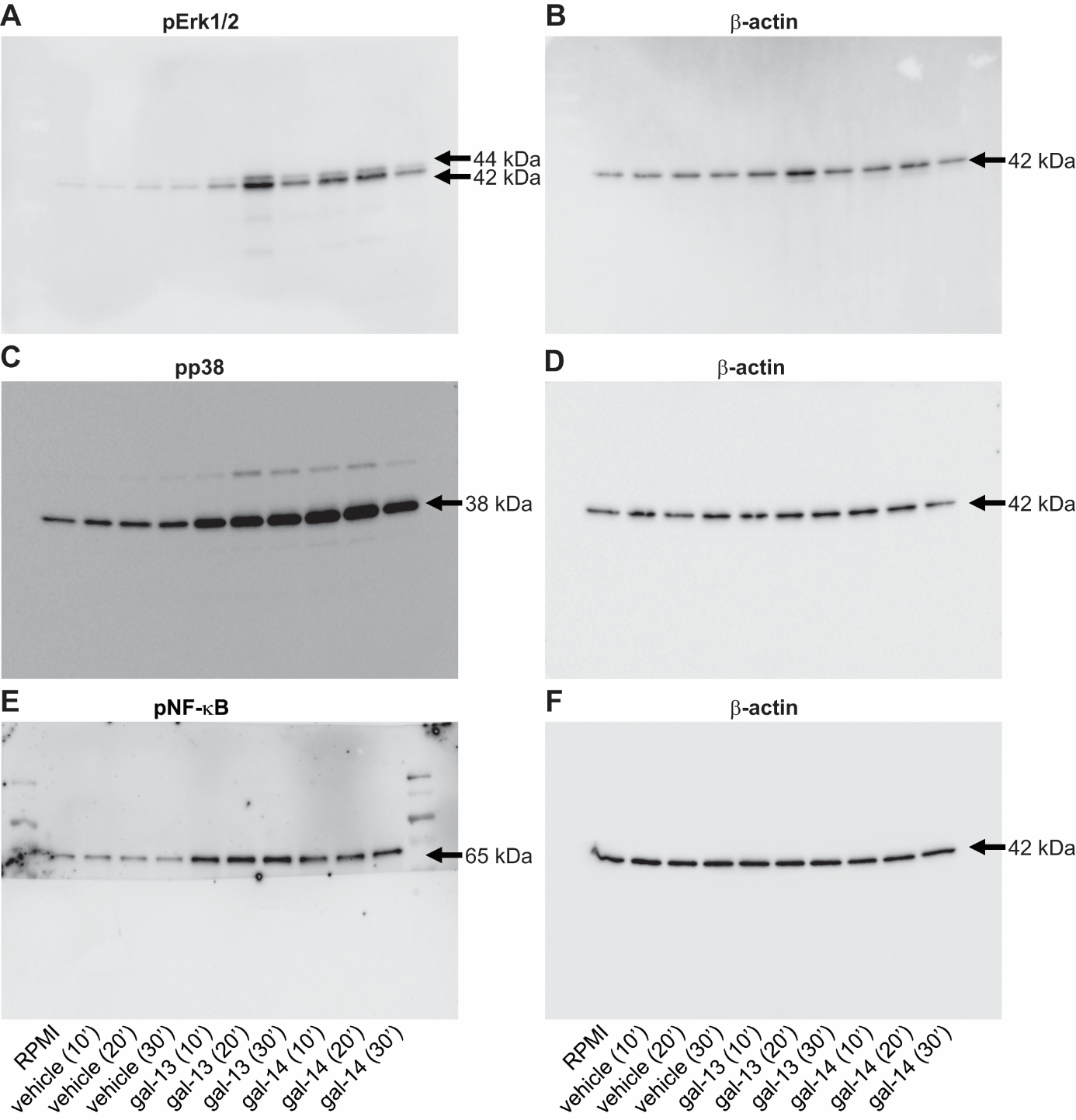


**Supplementary Figure 4. Galectin-13 and galectin-14 induce phosphorylation of Erk1/2, p38 MAPKs, and NF-κB.** The phosphorylation level of the mitogen-activated kinases Erk1/2 and p38, as well as transcription factor NF-κB was examined by Western blot. Freshly isolated PBMCs were incubated with vehicle, gal-13, or gal-14 for 10, 20, and 30 minutes. Serum-free medium (RPMI-1640) was used as absolute control. Representative membranes are displayed for Erk1/2 (**A**), p38 (**C**), and NF-κB (**E**), as well as for β-actin (**B, D, F**) as a loading control.
